# Taxonomy of Malagasy *Nesomyrmex brevicornis* species-group using conventional morphology-based approach

**DOI:** 10.3897/zookeys.616.9532

**Published:** 2016-09-12

**Authors:** Sándor Csősz, Brian L. Fisher

**Affiliations:** 1 Entomology, California Academy of Sciences, 55 Music Concourse Drive, San Francisco, CA 94118, U.S.A.

**Keywords:** Madagascar, taxonomy, morphometry, species delimitation, biogeography

## Abstract

Here we describe the diversity of the Malagasy *Nesomyrmex brevicornis*-group as the final installment of series describing the complete Malagasy *Nesomyrmex* fauna. In the current paper introduce the *Nesomyrmex brevicornis*-group, a newly outlined species group. We split this group from one of the four former groups, *Nesomyrmex madecassus*-group (sensu Csősz and Fisher 2015). The relatively small number of individuals available within the *Nesomyrmex brevicornis*-group relative to the large number of OTUs hampers the use of sophisticated statistical techniques to evaluate the morphological data. Instead, we assess the diversity of the *Nesomyrmex brevicornis*-group via a conventional morphology-based approach relying upon non-numeric characters (shape, sculpture, color etc.) recorded by simple eye inspection. Morphometric data are used as secondary data to confirm the most probable boundaries between species, but without employing statistical analyses. We rejected prior species hypotheses imposed by the primary approach if a non-overlapping range of a single index calculated from continuous numeric traits was found between two hypothesized species. Our methods render every species in this revisionary work separable via both salient features and morphometric traits. Our approach reveals the existence of nine morphologically distinct species, *Nesomyrmex brevicornis* **sp. n.**, *Nesomyrmex brunneus* **sp. n.**, *Nesomyrmex cingulatus* **sp. n.**, *Nesomyrmex edentates* **sp. n.**, *Nesomyrmex flavigaster* **sp. n.**, *Nesomyrmex longiceps* **sp. n.**, *Nesomyrmex minutus* **sp. n.**, *Nesomyrmex punctaticeps* **sp. n.**, and *Nesomyrmex sellaris* **sp. n.**. Geographic maps showing the distribution of each species are provided, along with an updated key to workers of Malagasy *Nesomyrmex* species groups that includes the newly outlined *Nesomyrmex brevicornis*-group.

## Introduction

The ant genus *Nesomyrmex* has been the subject of intensive systematic research in recent years, and contributes to efforts to explore the ant fauna of the Malagasy zoogeographical region, i.e. Madagascar and surrounding islands (Bolton 1994). Thanks to earlier explorations of the region’s biodiversity, our knowledge of the Malagasy *Nesomyrmex* fauna has increased considerably. The Malagasy members of the genus *Nesomyrmex* were split into four remarkable species groups by Csősz and Fisher (2015): *Nesomyrmex angulatus*-group (Csősz and Fisher 2016a), *Nesomyrmex hafahafa*-group (Csősz and Fisher 2015), *Nesomyrmex madecassus*-group (Csősz and Fisher 2016c) and *Nesomyrmex sikorai*-group (Csősz and Fisher 2015). Their classifications were revised using combined algorithmic approaches to evaluate numeric morphological data. The total species number has climbed to 24 of which 20 are new to science, underpinning earlier assumptions about the extreme species richness of the region.

In the current paper we complete the reexamination of Malagasy *Nesomyrmex* with a revision of the fifth species group, *Nesomyrmex brevicornis*-group, and by describing the nine species that are new to science. This group is subsequently split from the *Nesomyrmex madecassus*-group (sensu Csősz and Fisher 2015) based on several features, such as the remarkably deep mesopropodeal depression, the short scape (SL/CS: 0.49–0.72, in contrast to SL/CS of *madecassus*-group species: 0.72–0.83), the short and high petiole (PEL/CS: 0.34–0.49, in contrast to that of *madecassus*-group species: 0.44–0.58), the relatively long petiolar node (NOL/CS: 0.20–0.34, in contrast to that of *madecassus*-group species: 0.28–0.39, and the shorter mesosoma (ML/CS: 1.08–1.36, in contrast to that of *madecassus*-group species: 1.26–1.50) that distinguish workers of *brevicornis*-group from those of *madecassus*-group species.

All species of the *bervicornis*-group are arboreal; most nest in twigs or hollow branches, but some have very specialized associations with live stems or particular species of trees. *Nesomyrmex cingulatus* sp. n., for example, is known only from live stems from spiny thicket near Tsimanampetsotsa. *Nesomyrmex minutus* sp. n. is unique in nesting only in the stems of *Macphersonia gracilis* (Sapindaceae) and represents one of the few known ant plants in Madagascar. The specialized nesting habits of the *bervicornis*-group may explain why collections of this group are minimal compared to other *Nesomyrmex* species.

As the material available within this species group is insufficiently large for statistical evaluation, morphological diversity is assessed via evaluation of qualitative traits or trait-combination. Species are believed to have characteristic shapes, sizes, pigmentation patterns, sculpture, and other external features that may help in species separation and subsequent identification (Strauss and Bond 1990), hence qualitative morphological traits have often been the primary source for taxonomic studies (see e.g. Hita-Garcia and Fisher 2014, Snelling et al. 2014, Prebus 2015 etc.). An obvious advantage of this approach that it does not require large sample sizes to set species hypotheses. However, the often smaller number of available specimens hampers statistically supported hypothesis testing, and without morphometric analyses cryptic taxa may remain undiscovered. Overall, conventional morphology-based taxonomy remains the primary approach to outline species boundaries when small sample size precludes more complex research.

Qualitative evaluation of morphological data has revealed the existence of nine morphologically distinct species within *Nesomyrmex brevicornis* species-group in the Malagasy zoogeographical region, *Nesomyrmex brevicornis* sp. n., *Nesomyrmex brunneus* sp. n., *Nesomyrmex cingulatus* sp. n., *Nesomyrmex edentates* sp. n., *Nesomyrmex flavigaster* sp. n., *Nesomyrmex longiceps* sp. n., *Nesomyrmex minutus* sp. n., *Nesomyrmex punctaticeps* sp. n., and *Nesomyrmex sellaris* sp. n.. We provide a traditional, character-based key supported by simple numeric data based on body size ratios. Geographic maps showing the distribution of these species where populations of particular species were collected are also provided.

## Material and methods

In the present study, 21 continuous morphometric traits were recorded in 146 worker individuals belonging to 111 nest samples and 35 further single individuals collected in the Malagasy region.

The material is deposited in the following institutions, abbreviations after Evenhuis (2013): CASC (California Academy of Sciences, San Francisco, California, U.S.A.), MCZC (Museum of Comparative Zoology, Cambridge, Massachusetts, U.S.A.) and PSWC (Phil S. Ward’s collection, University of California Davis, Davis, California, U.S.A.). Type material and samples that were morphometrically investigated is presented in “Type material investigated” and “Material examined” sections in the format as follows: **CASENT code**, collection code, verbatim locality, longitude, latitude, elevation in meter, collector, date in MM.DD.YYYY format, (gender, abbreviation of depository). For genders (queen, worker, male) the first letter (Q, w, m) is used.

All images of specimens used in this study are available online on AntWeb (http://www.antweb.org). Images are linked to their specimens via the unique specimen code affixed to each pin (CASENT0027494). Online specimen identifiers follow this format: http://www.antweb.org/specimen/CASENT0027494.

Digital color montage images were created using a JVC KY-F75 digital camera and Syncroscopy Auto-Montage software (version 5.0), or a Leica DFC 425 camera in combination with the Leica Application Suite software (version 3.8). Distribution maps were generated in R (R Core Team 2015) via ‘phylo.to.map’ function using package phytools (Revell 2012).

Measurements were taken with a Leica MZ 12.5 stereomicroscope equipped with an ocular micrometer at a magnification of 100×. Measurements and indices are presented as arithmetic means with minimum and maximum values in parentheses. Body size dimensions are expressed in µm. Due to the abundance of worker individuals available relative to queen and male specimens, the present revision is based on worker caste only. Worker-based revision is further facilitated by the fact that the name-bearing type specimens of the vast majority of existing ant taxa belong to the worker caste. All measurements were made by the first author. For the definition of morphometric characters, earlier protocols (Csősz et al. 2015, Csősz and Fisher 2015, 2016a, 2016b) were considered. Explanations and abbreviations for measured characters are as follows:

CL Maximum cephalic length in median line. The head must be carefully tilted to the position providing the true maximum. Excavations of hind vertex and/or clypeus reduce CL.

CW Maximum width of the head. Includes compound eyes.

CWb Maximum width of head capsule without the compound eyes. Measured just posterior of the eyes.

CS Absolute cephalic size. The arithmetic mean of CL and CWb.

Cdep Antero-median clypeal depression. Maximum depth of the median clypeal depression on its anterior contour line as it appears in fronto-dorsal view.

EL Maximum diameter of the compound eye.

FRS Frontal carina distance. Distance of the frontal carinae immediately caudal of the posterior intersection points between frontal carinae and the torular lamellae. If these dorsal lamellae do not laterally surpass the frontal carinae, the deepest point of scape corner pits may be taken as the reference line. These pits occupy the inner corner of the scape base when the scape is directed caudally and produce a dark triangular shadow in the lateral frontal lobes immediately posterior to the dorsal lamellae of the scape joint capsule.

ML
**(Weber length)** Mesosoma length from caudalmost point of propodeal lobe to transition point between anterior pronotal slope and anterior pronotal shield. Preferentially measured in lateral view; if the transition point is not well defined, use dorsal view and take the center of the dark-shaded borderline between pronotal slope and pronotal shield as anterior reference point. In gynes: length from caudalmost point of propodeal lobe to the most distant point of steep anterior pronotal face.

MW Mesosoma width. In workers MW is defined as the longest width of the pronotum in dorsal view excluding the pronotal spines.

MPST Maximum distance from the center of the propodeal stigma to the anteroventral corner of the ventrolateral margin of the metapleuron.

NOH maximum height of the petiolar node. Measured in lateral view from the uppermost point of the petiolar node perpendicular to a reference line set from the petiolar spiracle to the imaginary midpoint of the transition between dorso-caudal slope and dorsal profile of caudal cylinder of the petiole.

NOL Length of the petiolar node. Measured in lateral view from the center of petiolar spiracle to dorso-caudal corner of caudal cylinder. Do not erroneously take as the reference point the dorso-caudal corner of the helcium, which is sometimes visible.

PEH maximum petiole height. The chord of the ventral petiolar profile at node level is the reference line perpendicular to which the maximum height of petiole is measured.

PEL Diagonal petiolar length in lateral view; measured from anterior corner of subpetiolar process to dorso-caudal corner of caudal cylinder.

PEW Maximum width of petiole in dorsal view. Nodal spines are not considered.

PoOC Postocular distance. Use a cross-scaled ocular micrometer and adjust the head to the measuring position of CL. Caudal measuring point: median occipital margin; frontal measuring point: median head at the level of the posterior eye margin.

PPH Maximum height of the postpetiole in lateral view. Measured perpendicularly to a line defined by the linear section of the segment border between dorsal and ventral petiolar sclerite.

PPL Postpetiole length. The longest anatomical line that is perpendicular to the posterior margin of the postpetiole and is between the posterior postpetiolar margin and the anterior postpetiolar margin.

PPW Postpetiole width. Maximum width of postpetiole in dorsal view.

SL Scape length. Maximum straight line scape length excluding the articular condyle.

SPST Spine length. Distance between the center of propodeal stigma and spine tip. The stigma center refers to the midpoint defined by the outer cuticular ring but not to the center of the real stigma opening, which may be positioned eccentrically.

SPTI Apical spine distance. The distance of spine tips in dorsal view; if spine tips are rounded or truncated, the centers of spine tips are taken as reference points.

In verbal descriptions of taxa based on external morphological traits, recent taxonomic papers (Csősz and Fisher 2015, 2016) were considered. Definitions of surface sculpturing are linked to Harris (1979). Body size is given in µm, and means of morphometric ratios as well as minimum and maximum values are given in parentheses with up to three digits. Inclination of pilosity and cuticular spines is given in degrees. Definitions of species-groups as well as descriptions of species are surveyed in alphabetic order.

**Hypothesis formation.** The present paper aims to reveal diversity of *Nesomyrmex brevicornis* species-group, including a number of very rare species represented only by three to four individuals. This high level of asymmetry in data, coupled with the extremely low average number of individuals per species, prevents employment of statistical analyses in hypothesis formation and testing. Instead, the conventional morphology-based approach (i.e., individuals are sorted into clusters using simple eye inspection based on salient morphological features, e.g., shape, sculpture etc.) is used as the primary technique for recognizing species. Morphometric data are given for each species as secondary data for confirmation and in order to improve identification.

## Results

Based on the applied conventional morphology-based approach, nine remarkably different morphologies are recognized to be considered species. These species can also be characterized by, and separated from each other using, at least one non-overlapping body-size ratio. Morphometric indices are provided for all species in Table 1. To the best of our knowledge, all are endemic to Madagascar.

**Table 1.** Mean of morphometric ratios calculated species-wise on individual level. Morphometric traits are divided by absolute cephalic size (CS), ±SD are provided in the upper row, minimum and maximum values are given in parentheses in the lower row.

	*brevicornis* (n = 3)	*brunneus* (n = 36)	*cingulatus* (n = 26)	*edentatus* (n = 3)	*flavigaster* (n = 14)	*longiceps* (n = 4)	*minutus* (n = 29)	*punctaticeps* (n = 17)	*sellaris* (n = 15)
**CS**	506 ±33	721 ±58	459 ±25	546 ±13	637 ±51	531 ±36	472 ±31	473 ±30	449 ±36
	[472, 536]	[620, 808]	[419, 515]	[535, 560]	[546, 740]	[478, 556]	[405, 549]	[424, 549]	[410, 557]
**CL/CW**	1.32 ±0.05	1.12 ±0.03	1.34 ±0.04	1.19 ±0.00	1.21 ±0.02	1.42 ±0.02	1.29 ±0.05	1.17 ±0.04	1.16 ±0.04
	[1.28, 1.37]	[1.07, 1.19]	[1.26, 1.39]	[1.18, 1.19]	[1.16, 1.25]	[1.40, 1.44]	[1.17, 1.36]	[1.11, 1.23]	[1.09, 1.25]
**CL/CWb**	1.33 ±0.02	1.14 ±0.02	1.37 ±0.04	1.20 ±0.01	1.24 ±0.02	1.43 ±0.02	1.29 ±0.05	1.20 ±0.04	1.18 ±0.03
	[1.32, 1.35]	[1.09, 1.21]	[1.30, 1.45]	[1.19, 1.21]	[1.21, 1.29]	[1.42, 1.46]	[1.20, 1.36]	[1.14, 1.26]	[1.13, 1.25]
**POoC/CL**	0.52 ±0.01	0.44 ±0.03	0.51 ±0.01	0.48 ±0.01	0.43 ±0.01	0.44 ±0.01	0.52 ±0.01	0.49 ±0.01	0.51 ±0.01
	[0.51, 0.52]	[0.39, 0.47]	[0.49, 0.52]	[0.47, 0.49]	[0.41, 0.45]	[0.42, 0.45]	[0.51, 0.54]	[0.47, 0.51]	[0.49, 0.53]
**FRS/CS**	0.36 ±0.02	0.33 ±0.01	0.31 ±0.01	0.30 ±0.01	0.34 ±0.01	0.26 ±0.01	0.35 ±0.02	0.33 ±0.01	0.29 ±0.01
	[0.34, 0.37]	[0.31, 0.35]	[0.30, 0.33]	[0.29, 0.31]	[0.33, 0.36]	[0.25, 0.28]	[0.30, 0.39]	[0.32, 0.35]	[0.27, 0.30]
**SL/CS**	0.54 ±0.01	0.64 ±0.02	0.63 ±0.01	0.66 ±0.01	0.62 ±0.02	0.63 ±0.02	0.57 ±0.02	0.66 ±0.02	0.69 ±0.02
	[0.53, 0.55]	[0.60, 0.67]	[0.60, 0.65]	[0.65, 0.67]	[0.59, 0.66]	[0.61, 0.65]	[0.49, 0.61]	[0.62, 0.70]	[0.65, 0.72]
**EL/CS**	0.25 ±0.01	0.22 ±0.01	0.24 ±0.01	0.25 ±0.01	0.23 ±0.01	0.22 ±0.01	0.25 ±0.01	0.24 ±0.01	0.24 ±0.01
	[0.24, 0.26]	[0.20, 0.24]	[0.22, 0.26]	[0.23, 0.26]	[0.21, 0.25]	[0.21, 0.23]	[0.22, 0.28]	[0.22, 0.26]	[0.23, 0.26]
**MW/CS**	0.59 ±0.02	0.60 ±0.01	0.55 ±0.01	0.56 ±0.02	0.59 ±0.01	0.53 ±0.03	0.56 ±0.01	0.61 ±0.02	0.58 ±0.01
	[0.57, 0.60]	[0.56, 0.62]	[0.52, 0.57]	[0.54, 0.58]	[0.58, 0.61]	[0.50, 0.55]	[0.54, 0.58]	[0.58, 0.64]	[0.56, 0.59]
**PEW/CS**	0.23 ±0.02	0.20 ±0.01	0.22 ±0.01	0.20 ±0.01	0.22 ±0.01	0.19 ±0.00	0.22 ±0.01	0.22 ±0.01	0.21 ±0.01
	[0.21, 0.25]	[0.19, 0.22]	[0.21, 0.24]	[0.20, 0.22]	[0.20, 0.22]	[0.19, 0.19]	[0.20, 0.23]	[0.21, 0.24]	[0.20, 0.23]
**PPW/CS**	0.31 ±0.01	0.26 ±0.01	0.32 ±0.01	0.28 ±0.01	0.31 ±0.01	0.27 ±0.01	0.33 ±0.01	0.34 ±0.02	0.29 ±0.01
	[0.30, 0.33]	[0.24, 0.30]	[0.28, 0.35]	[0.27, 0.28]	[0.29, 0.33]	[0.26, 0.28]	[0.29, 0.35]	[0.30, 0.36]	[0.26, 0.31]
**ML/CS**	1.30 ±0.01	1.18 ±0.03	1.30 ±0.02	1.27 ±0.04	1.21 ±0.02	1.14 ±0.03	1.29 ±0.03	1.26 ±0.02	1.23 ±0.02
	[1.29, 1.31]	[1.08, 1.23]	[1.26, 1.36]	[1.23, 1.31]	[1.17, 1.23]	[1.12, 1.19]	[1.23, 1.36]	[1.22, 1.32]	[1.19, 1.26]
**PEL/CS**	0.42 ±0.01	0.38 ±0.02	0.46 ±0.02	0.41 ±0.01	0.42 ±0.01	0.39 ±0.01	0.45 ±0.02	0.43 ±0.01	0.41 ±0.02
	[0.41, 0.43]	[0.34, 0.42]	[0.41, 0.49]	[0.39, 0.42]	[0.40, 0.43]	[0.38, 0.40]	[0.42, 0.47]	[0.41, 0.47]	[0.38, 0.44]
**NOL/CS**	0.26 ±0.01	0.23 ±0.01	0.32 ±0.01	0.26 ±0.01	0.23 ±0.01	0.24 ±0.02	0.26 ±0.02	0.28 ±0.01	0.25 ±0.02
	[0.25, 0.27]	[0.20, 0.25]	[0.28, 0.34]	[0.25, 0.26]	[0.22, 0.25]	[0.22, 0.26]	[0.22, 0.29]	[0.26, 0.31]	[0.22, 0.27]
**MPST/CS**	0.42 ±0.01	0.39 ±0.01	0.41 ±0.01	0.40 ±0.02	0.41 ±0.01	0.37 ±0.01	0.41 ±0.01	0.40 ±0.01	0.40 ±0.01
	[0.41, 0.43]	[0.37, 0.41]	[0.37, 0.43]	[0.38, 0.41]	[0.40, 0.41]	[0.36, 0.38]	[0.38, 0.45]	[0.39, 0.42]	[0.38, 0.43]
**PEH/CS**	0.28 ±0.02	0.27 ±0.01	0.27 ±0.01	0.27 ±0.01	0.31 ±0.01	0.24 ±0.01	0.31 ±0.02	0.28 ±0.01	0.27 ±0.01
	[0.26, 0.30]	[0.24, 0.30]	[0.26, 0.30]	[0.25, 0.28]	[0.29, 0.33]	[0.22, 0.25]	[0.26, 0.34]	[0.27, 0.31]	[0.25, 0.29]
**NOH/CS**	0.10 ±0.01	0.12 ±0.02	0.10 ±0.01	0.10 ±0.01	0.13 ±0.00	0.09 ±0.01	0.13 ±0.01	0.11 ±0.01	0.09 ±0.01
	[0.08, 0.10]	[0.10, 0.15]	[0.09, 0.12]	[0.09, 0.11]	[0.12, 0.14]	[0.08, 0.09]	[0.11, 0.15]	[0.09, 0.13]	[0.07, 0.11]
**PPH/CS**	0.25 ±0.01	0.23 ±0.02	0.27 ±0.01	0.22 ±0.01	0.28 ±0.01	0.21 ±0.01	0.26 ±0.01	0.28 ±0.01	0.22 ±0.01
	[0.24, 0.26]	[0.18, 0.27]	[0.25, 0.30]	[0.22, 0.23]	[0.26, 0.30]	[0.21, 0.22]	[0.23, 0.29]	[0.26, 0.29]	[0.19, 0.24]
**PPL/CS**	0.20 ±0.02	0.18 ±0.02	0.29 ±0.01	0.19 ±0.01	0.21 ±0.01	0.21 ±0.00	0.24 ±0.01	0.28 ±0.02	0.21 ±0.01
	[0.18, 0.22]	[0.15, 0.21]	[0.26, 0.31]	[0.18, 0.21]	[0.18, 0.23]	[0.21, 0.21]	[0.22, 0.26]	[0.26, 0.31]	[0.19, 0.23]

### Key to species-groups

**Table:** 

1	Anterodorsal spines on petiolar node present	***hafahafa* -group**
–	Anterodorsal spines on petiolar node absent	**2**
2	Petiolar node globular in dorsal view, sides straongly convex. Postocular distance vs. petiole width (PoOc/PEW): ≤1.2 [0.72, 1.17]. Sculpture of head, mesosoma, petiole and postpetiole areolate, appears dull. Main sculpture homogenous reticulate, or rugoso-reticulate	***angulatus* -group**
–	Petiolar node long and narrow in dorsal view, sides nearly parallel. Postocular distance vs. petiole width (PoOc/PEW): ≥1.2 [1.2, 2.01]. Ground sculpture on head, dorsum of mesosoma, petiole and postpetiole often, but not exclusively inconspicuous, shiny. Main sculpture variable, coarse to absent	**3**
3	Petiolar node in lateral view higher, (MPST/NOH): 2.41 [1.89, 2.87], propodeal spines moderately long, always present, mesopropodeal depression conspicuous, deep	***sikorai* -group**
–	Petiolar node in lateral view lower, (MPST/NOH): 3.54 [2.71, 5.63], propodeal spines very short to absent, mesopropodeal depression variable	**4**
4	Mesopropodeal depression absent (except for *Nesomyrmex gibber* where a mesothoracic hump is conspicuous). Scape longer (SL/CS): 0.72–0.83. Petiole longer (PEL/CS): 0.44–0.58)	***madecassus* -group**
–	Mesopropodeal depression present and conspicuous (often shallow in *Nesomyrmex flavigaster*). Scape shorter (SL/CS): 0.49–0.72. Petiole shorter and higher (PEL/CS): 0.34–0.49	***brevicornis* -group**

### Synopsis of Malagasy members of the *Nesomyrmex brevicornis* species group

*brevicornis* Csősz & Fisher, sp. n.

*brunneus* Csősz & Fisher, sp. n.

*cingulatus* Csősz & Fisher, sp. n.

*edentatus* Csősz & Fisher, sp. n.

*flavigaster* Csősz & Fisher, sp. n.

*longiceps* Csősz & Fisher, sp. n.

*minutus* Csősz & Fisher, sp. n.

*punctaticeps* Csősz & Fisher, sp. n.

*sellaris* Csősz & Fisher, sp. n.

### Key to workers of the *Nesomyrmex madecassus* group species

**Table:** 

1	Head and mesosoma dark brown to black	**2** (*brevicornis*, *brunneus*, *edentatus*, *flavigaster*)
–	Head and mesosoma yellow	**5** (*cingulatus*, *longiceps*, *minutus*, *punctaticeps*, *sellaris*)
2	Gaster pale yellow, the rest of the body dark brown to black (Fig. 1)	***flavigaster***
–	Body—including gaster—concolorous, dark brown to black (Fig. 2)	**3** (*brevicornis*, *brunneus*, *edentatus*)
3	Large species, cephalic size (CS) > 620 µm [620, 808]	***brunneus***
–	Smaller species, cephalic size (CS) < 560 µm [472, 560]	**4** (*brevicornis*, *edentatus*)
4	Head feebly imbricate, appears shiny (Fig. 3). Scape longer (SL/CS) > 0.65 [0.65, 0.67]	***edentatus***
–	Head coarsely imbricate, appears dull (Fig. 4). Scape shorter (SL/CS) < 0.55 [0.53, 0.55]	***brevicornis***
5	First gaster tergite brown, conspicuously darker than the rest of the body (Fig. 5)	**6** (*cingulatus*, *minutus*)
–	Body concolorous, gaster light yellow	**7** (*longiceps*, *punctaticeps*, *sellaris*)
6	On the median area of frons foveae around setal pits larger (Fig. 6), the largest diameter of pits 15–20 µm. Frontal carina distance (FRS/CS): 0.31 [0.30, 0.33], scape length (SL/CS): 0.63 [0.60, 0.65]	***cingulatus***
–	On the median area of frons foveae around setal pits inconspicuous (Fig. 7), the largest diameter of pits ≤ 10 µm. Frontal carina distance (FRS/CS): 0.36 [0.33, 0.39], scape length (SL/CS): 0.57 [0.53, 0.61]	***minutus***
7	Head longer (CL/CWb) >1.40 [1.42, 1.46], postocular area relatively shorter (PoOC/CL) < 0.45 [0.42, 0.45]	***longiceps***
–	Head shorter (CL/CWb) < 0.30 [1.14, 1.26], postocular area relatively longer (PoOC/CL) > 0.47 [0.47, 0.53]	**8** (*punctaticeps*, *sellaris*)
8	Frontal carina distance larger (FRS/CS) > 0.33 [0.32, 0.35]. On the median area of frons foveae around setal pits larger (Fig. 8), the largest diameter of pits 10–15 µm	***punctaticeps***
–	Frontal carina distance smaller (FRS/CS) < 0.29 [0.27, 0.30]. On the median area of frons setal pits are not embedded by foveae (Fig. 9)	***sellaris***

**Figures 1–4.**
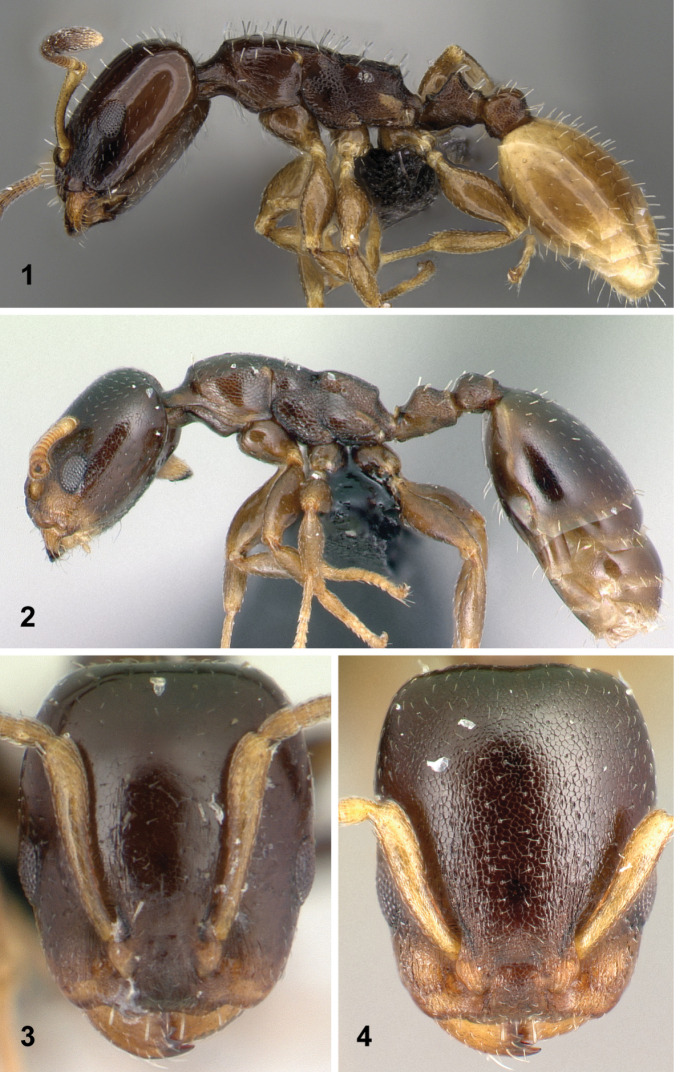
Diagnostic traits for black *Nesomyrmex brevicornis*-group workers. Lateral view of mesosoma, petiole, postpetiole, and gaster of *Nesomyrmex flavigaster* sp. n. (**1**), lateral view of mesosoma, petiole, postpetiole, and gaster of *Nesomyrmex brunneus* sp. n. (**2**), frontal view of head of *Nesomyrmex edentatus* sp. n. (**3**), frontal view of head of *Nesomyrmex brevicornis* sp. n. (**4**). For details see main text.

**Figures 5–9.**
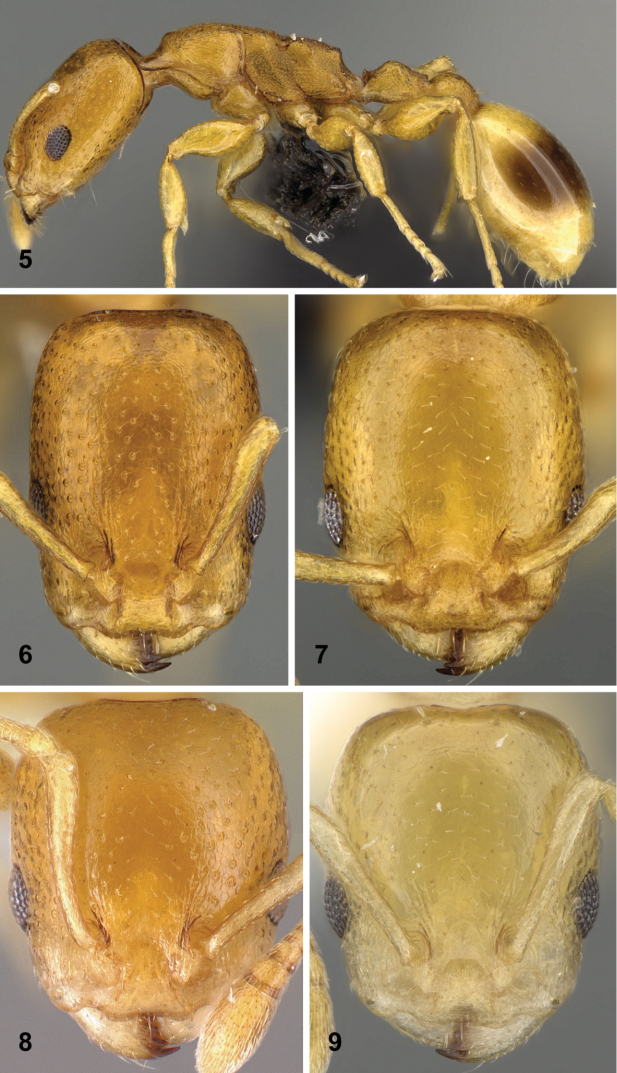
Diagnostic traits for yellow *Nesomyrmex brevicornis*-group workers. Lateral view of mesosoma, petiole, postpetiole and gaster of *Nesomyrmex cingulatus* sp. n. (**5**), frontal view of head of *Nesomyrmex cingulatus* sp. n. (**6**), frontal view of head of *Nesomyrmex minutus* sp. n. (**7**), frontal view of head of *Nesomyrmex punctaticeps* sp. n. (**8**), frontal view of head of *Nesomyrmex sellaris* sp. n. (**9**). For details see main text.

#### Nesomyrmex brevicornis

Taxon classificationAnimaliaHymenopteraFormicidae

Csősz & Fisher sp. n.

http://zoobank.org/0DA355E3-6F8F-41AC-B04D-CED946D108B7

**Figures 10–12.**
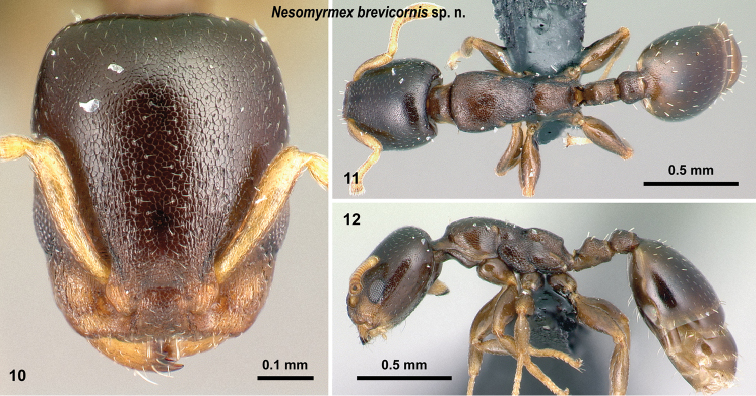
*Nesomyrmex brevicornis* sp. n. holotype worker (CASENT0027494). Head in full-face view (**10**), dorsal view of the body (**11**), lateral view of the body (**12**).

**Figures 13.**
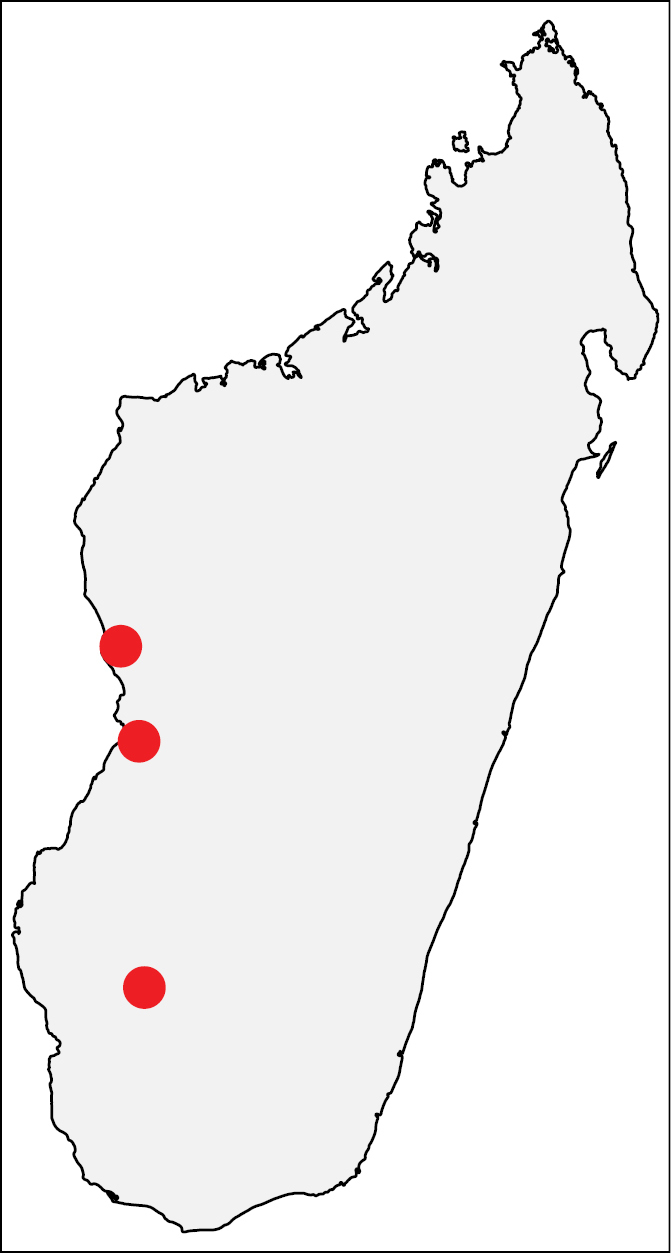
Geographic distribution of *Nesomyrmex brevicornis* sp. n.. The known localities of *Nesomyrmex brevicornis* sp. n. in Madagascar.

Figs 10–12 , 13 , Table 1

##### Type material investigated.

**Holotype: CASENT0027494**, collection code: BLF07507, Parc National de Zombitse, 19.8 km 84° E Sakaraha, N -22.84333°, 44.71°, alt 770 m, Fisher, Griswold et al., 02.05.2003, (1w, CASC, CASENT0027494);

**Paratypes: CASENT0195104**, collection code: DMO-CS7, 48 km ENE Morondava, Kirindy, -20.06667°, 44.65°, alt 30 m, D.M.Olson, 01.05.1991, (1w, PSWC); **CASENT0482280**, collection code: BLF04508, Forêt de Tsimembo, 11.0 km 346° NNW Soatana, N -18.99528°, 44.4435°, alt 50 m, Fisher-Griswold Arthropod Team, 11.21.2001, (1w, CASC);

##### Etymology.

The name (brevicornis) refers to the short antennal scape that distinguishes this species from other taxa treated in this revisionary work.

##### Diagnosis in key.

**Description of workers.** Body color: dark brown; black. Body color pattern: concolorous. Absolute cephalic size: 506 [472, 536]. Cephalic length vs. maximum width of head capsule (CL/CWb): 1.33 [1.32, 1.35]. Postocular distance vs. cephalic length (PoOc/CL): 0.52 [0.51, 0.52]. Postocular sides of cranium contour frontal view orientation: converging posteriorly. Postocular sides of cranium contour frontal view shape: broadly convex. Vertex contour line in frontal view shape: straight. Vertex sculpture: main sculpture absent, ground sculpture areolate. Setal pits on head dorsum: inconspicuous with obscure demarcation. Gena contour line in frontal view shape: convex. Genae contour from anterior view orientation: converging. Gena sculpture: ground sculpture areolate, main sculpture absent. Concentric carinae laterally surrounding antennal foramen: present. Eye length vs. absolute cephalic size (EL/CS): 0.25 [0.24, 0.26]. Frontal carina distance vs. absolute cephalic size (FRS/CS): 0.36 [0.34, 0.37]. Longitudinal carinae on median region of frons: absent. Smooth median region on frons: present. Antennomere count: 12. Scape length vs. absolute cephalic size (SL/CS): 0.54 [0.53, 0.55]. Median clypeal notch: present. Ground sculpture of submedian area of clypeus: present. Median carina of clypeus: absent. Metanotal depression: present. Dorsal region of mesosoma sculpture: ground sculpture areolate, main sculpture absent. Lateral region of pronotum sculpture: ground sculpture areolate, main sculpture absent. Mesopleuron sculpture: ground sculpture areolate, main sculpture absent. Metapleuron sculpture: ground sculpture areolate, main sculpture absent. Petiole width vs. absolute cephalic size (PEW/CS): 0.23 [0.21, 0.25]. Dorsal region of petiole sculpture: ground sculpture areolate, main sculpture absent. Postpetiole width vs. absolute cephalic size (PPW/CS): 0.31 [0.30, 0.33]. Dorsal region of postpetiole sculpture: ground sculpture areolate, main sculpture absent. Surface of first gastral tergite: continuously imbricate.

##### Distribution.

This species is known to occur in the southwestern part of Madagascar from 30–770 m (Fig. 13). The known samples are collected from sifted litter (leaf mold, rotten wood) in tropical dry forest.

#### Nesomyrmex brunneus

Taxon classificationAnimaliaHymenopteraFormicidae

Csősz & Fisher sp. n.

http://zoobank.org/AE916393-5BC3-4511-8D5A-DF045F391A45

**Figures 14–16.**
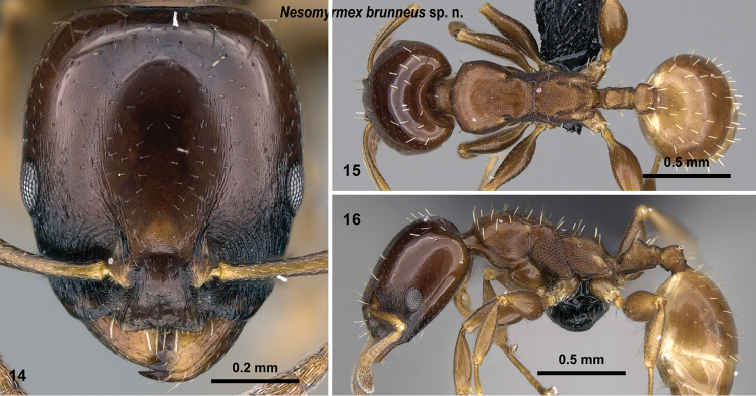
*Nesomyrmex brunneus* sp. n. holotype worker (CASENT0919844). Head in full-face view (**14**), dorsal view of the body (**15**), lateral view of the body (**16**).

**Figure 17.**
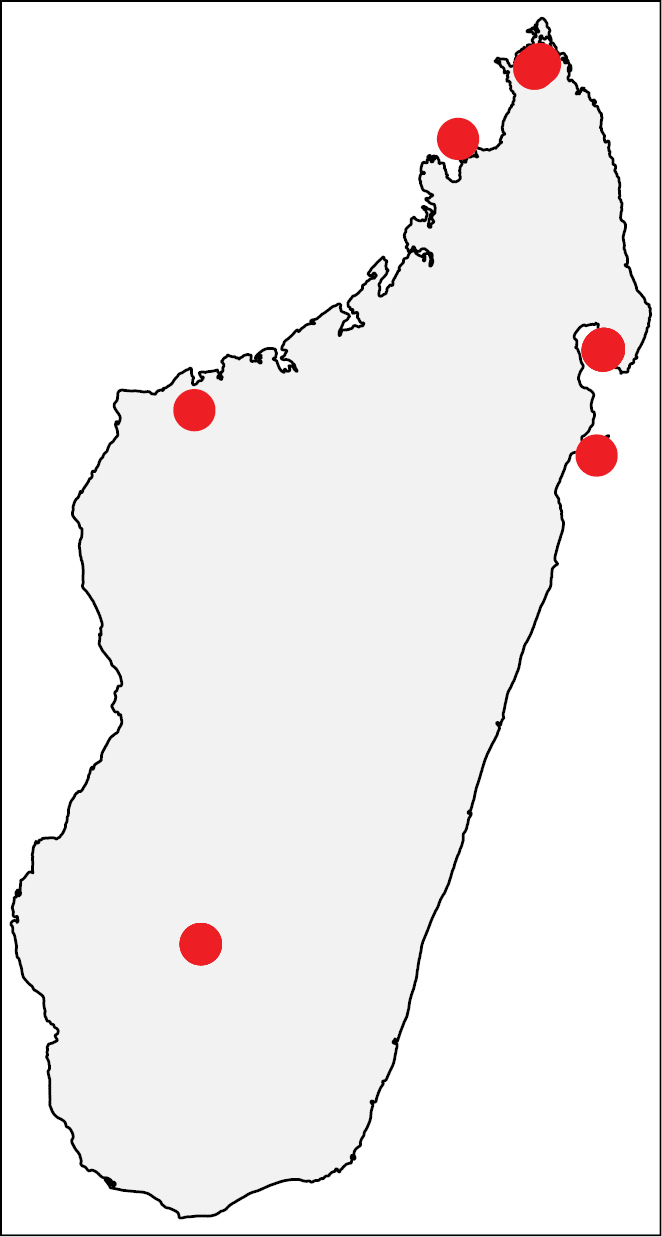
Geographic distribution of *Nesomyrmex brunneus* sp. n.. The known localities of *Nesomyrmex brunneus* sp. n. shown in Madagascar.

Figs 14–16 , 17 , Table 1

##### Type material investigated.

**Holotype**: CASENT0919844, collection code: ANTC1302, P.N. Masoala, 40 km 154° SSE Maroantsetra, -15.72667°, 49.95667°, alt 150 m, A. Dejean et al., 10.14.2001 (1w, CASC, CASENT0919844);

**Paratypes**: eight workers and two gynes from the same locality under CASENT codes: CASENT0004957, collection code: ANTC1302, (2w, CASC); CASENT0004958, collection code: ANTC1302, (1Q, CASC); CASENT0004959, collection code: ANTC1302, (1Q, CASC); CASENT0008660, collection code: ANTC1302, (2w, CASC, CASENT0008660); CASENT0008661, collection code: ANTC1302, (2w, CASC); CASENT0008662, collection code: ANTC1302, (2w, CASC);

##### Material examined.

**MADAGASCAR: CASENT0066844**, collection code: BLF13087, Ile Sainte Marie, Forêt Kalalao, 9.9 km 34° Ambodifotatra, -16.9225°, 49.88733°, alt 100 m, B.L.Fisher et al., 11.26.2005, (1w, CASC); **CASENT0082404**, collection code: MA-01-01D-07, Parc National Montagne d’Ambre [Petit Lac road], -12.52028°, 49.17917°, alt 1125 m, R. Harin’Hala, 04.05.2001, (1w, CASC, CASENT0082404); **CASENT0083199**, collection code: MA-01-01D-09, Parc National Montagne d’Ambre [Petit Lac road], -12.52028°, 49.17917°, alt 1125 m, R. Harin’Hala, 04.26.2001, (1w, CASC); **CASENT0107525**, collection code: BLF11668, Nosy Be Airport, 10.3 km 30° Hell Ville, -13.32017°, 48.31083°, alt 25 m, B.L.Fisher, 12.11.2004, (1w, CASC, CASENT0107525); **CASENT0138300**, collection code: BLF20144, Manerinerina, 76.6 km N Antsohihy, -14.10744°, 48.11046°, alt 247 m, B.L.Fisher et al., 04.11.2008, (1w, CASC, CASENT0138300); **CASENT0195102**, collection code: PSW11939, 9km NNW Ranohira, P.N. Isalo, -22.48333°, 45.38333°, alt 800 m, P.S.Ward, 2.16.1993, (2w, PSWC); **CASENT0195103**, collection code: PSW11943, 9km NNW Ranohira, P.N. Isalo, -22.48333°, 45.38333°, alt 800 m, P.S.Ward, 02.16.1993, (2w, PSWC); **CASENT0418178**, collection code: BLF04708, Parc National de Masoala, 39.4 km 150° SSE Maroantsetra, -15.71°, 49.97°, alt 200 m, B.L.Fisher, H.J.Ratsirarson, 11.28.2001, (2w, CASC); **CASENT0418217**, collection code: BLF04724, Parc National de Masoala, 39.4 km 150° SSE Maroantsetra, -15.71°, 49.97°, alt 200 m, B.L.Fisher, H.J.Ratsirarson, 11.28.2001, (1w, CASC); **CASENT0418218**, collection code: BLF04723, Parc National de Masoala, 39.4 km 150° SSE Maroantsetra, -15.71°, 49.97°, alt 200 m, B.L.Fisher, H.J.Ratsirarson, 11.28.2001, (1w, CASC); **CASENT0418234**, collection code: BLF04696, Tampolo, Masoala Peninsula, 40.4 km 154° SSE Maroantsetra, -15.73°, 49.96°, alt 30 m, B.L.Fisher, H.J.Ratsirarson, 11.28.2001, (2w, CASC); **CASENT0418244**, collection code: BLF04711, Parc National de Masoala, 39.4 km 150° SSE Maroantsetra, -15.71°, 49.97°, alt 200 m, B.L.Fisher, H.J.Ratsirarson, 11.28.2001, (2w, CASC); **CASENT0418285**, collection code: BLF04710, Parc National de Masoala, 39.4 km 150° SSE Maroantsetra, -15.71°, 49.97°, alt 200 m, B.L.Fisher, H.J.Ratsirarson, 11.28.2001, (2w, CASC); **CASENT0428256**, collection code: BLF02660, Réserve Spéciale d’Ambre, 3.5 km 235° SW Sakaramy, -12.46889°, 49.24217°, alt 325 m, Fisher, Griswold et al., 01.26.2001, (1w, CASC); **CASENT0488569**, collection code: BLF06584, Parc National de Namoroka, 16.9 km 317° NW Vilanandro, -16.40667°, 45.31°, alt 100 m, Fisher, Griswold et al., 11.12.2002, (1w, CASC, CASENT0488569); **MCZENT0576251** 9km NNW Ranohira, P.N. Isalo, -22.48333°, 45.38333°, alt 800 m, P.S.Ward, 02.16.1993, (2w, MCZC); **MCZENT0576252**, collection code: 11939, 9km NNW Ranohira, P.N. Isalo, -22.48333°, 45.38333°, alt 800 m, P.S.Ward, 02.16.1993, (2w, MCZC); **MCZENT0576253**, collection code: 11939, 9km NNW Ranohira, P.N. Isalo, -22.48333°, 45.38333°, alt 800 m, P.S.Ward, 02.16.1993, (2w, MCZC).

##### Etymology.

This name (brunneus) refers to the concolorous dark brown appearance of the workers of this species.

##### Diagnosis in key.

**Description of workers.** Body color: dark brown; black. Body color pattern: concolorous. Absolute cephalic size: 721 [620, 808]. Cephalic length vs. maximum width of head capsule (CL/CWb): 1.14 [1.09, 1.21]. Postocular distance vs. cephalic length (PoOc/CL): 0.44 [0.39, 0.47]. Postocular sides of cranium contour frontal view orientation: converging posteriorly. Postocular sides of cranium contour frontal view shape: broadly convex. Vertex contour line in frontal view shape: straight. Vertex sculpture: main sculpture absent, ground sculpture absent, smooth and shiny. Setal pits on head dorsum: inconspicuous with obscure demarcation. Gena contour line in frontal view shape: convex. Genae contour from anterior view orientation: converging; strongly converging. Gena sculpture: ground sculpture areolate, main sculpture absent. Concentric carinae laterally surrounding antennal foramen: present. Eye length vs. absolute cephalic size (EL/CS): 0.22 [0.20, 0.24]. Frontal carina distance vs. absolute cephalic size (FRS/CS): 0.33 [0.31, 0.35]. Longitudinal carinae on median region of frons count: absent. Smooth median region on frons: present. Antennomere count: 12. Scape length vs. absolute cephalic size (SL/CS): 0.64 [0.60, 0.67]. Median clypeal notch: present. Ground sculpture of submedian area of clypeus: present. Median carina of clypeus: absent. Metanotal depression: present. Dorsal region of mesosoma sculpture: ground sculpture areolate, main sculpture absent. Lateral region of pronotum sculpture: ground sculpture areolate, main sculpture absent. Mesopleuron sculpture: ground sculpture areolate, main sculpture absent. Metapleuron sculpture: ground sculpture areolate, main sculpture absent. Petiole width vs. absolute cephalic size (PEW/CS): 0.20 [0.19, 0.22]. Dorsal region of petiole sculpture: ground sculpture areolate, main sculpture absent. Postpetiole width vs. absolute cephalic size (PPW/CS): 0.26 [0.24, 0.29]. Dorsal region of postpetiole sculpture: ground sculpture areolate, main sculpture absent. Surface of first gastral tergite: continuously imbricate.

##### Distribution.

This species occurs predominantly in rainforests of northern Madagascar from 25–800 m. The only southern population is reported from a rain forest of Isalo National Park (Fig. 17). The known samples are collected from canopy: canopy moss and leaf litter or rotten pockets of live branches.

#### Nesomyrmex cingulatus

Taxon classificationAnimaliaHymenopteraFormicidae

Csősz & Fisher sp. n.

http://zoobank.org/E91068C9-E12A-4824-A56A-DF1CA89F2A79

**Figures 18–20.**
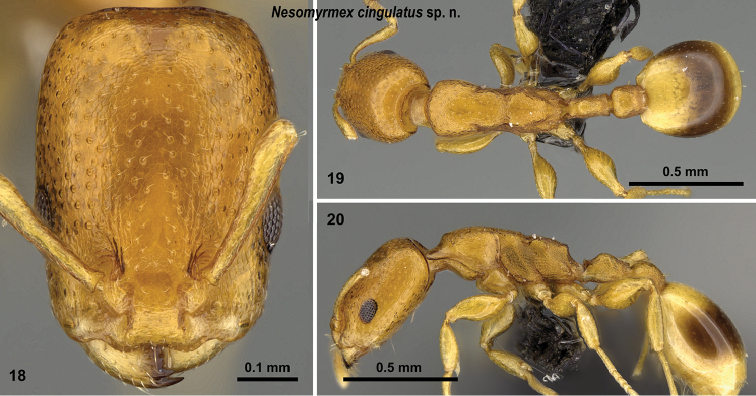
*Nesomyrmex cingulatus* sp. n. holotype worker (CASENT0919845). Head in full-face view (**18**), dorsal view of the body (**19**), lateral view of the body (**20**).

**Figures 21.**
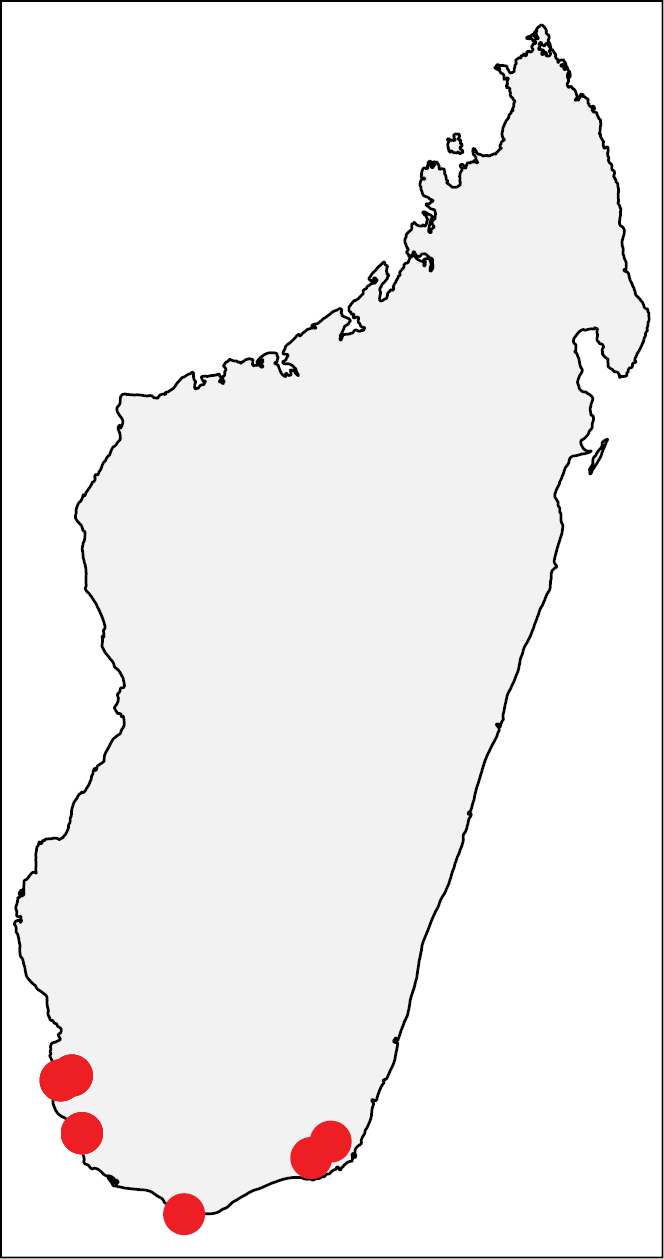
Geographic distribution of *Nesomyrmex cingulatus* sp. n.. The known localities of *Nesomyrmex cingulatus* sp. n. in Madagascar.

Figs 18–20 , 21 , Table 1

##### Type material investigated.

**Holotype: CASENT0919845**, collection code: BLF06205, Parc National de Tsimanampetsotsa, Mitoho Cave, 6.4 km 77° ENE Efoetse, 17.4 km 170° S Beheloka, -24.04722°, 43.75317°, alt 40 m, Fisher-Griswold Arthropod Team, 03.18.2002, (1w, CASC, CASENT0919845);

**Paratypes**: 22 workers and a gyne from the same locality under CASENT codes: **CASENT0455751**, collection code: BLF06204, (2w, CASC); **CASENT0455757**, collection code: BLF06205, (2w, CASC); **CASENT0455756**, collection code: BLF06205, (3w, CASC); **CASENT0455755**, collection code: BLF06205, (3w, CASC); **CASENT0455759**, collection code: BLF06205, (1Q, CASC); **CASENT0455750**, collection code: BLF06204, (3w, CASC); **CASENT0455752**, collection code: BLF06204, (3w, CASC); **CASENT0455753**, collection code: BLF06204, (3w, CASC); **CASENT0455754**, collection code: BLF06204, (3w, CASC);

##### Material examined.

**MADAGASCAR: CASENT0003993**, collection code: BLF05832, Mahafaly Plateau, 6.2 km 74° ENE Itampolo, -24.65361°, 43.99667°, alt 80 m, Fisher-Griswold Arthropod Team, 02.21.2002, (2w, CASC); **CASENT0061872**, collection code: MA-02-20-12, Tsimelahy - Parcel II, Andohahela National Park, transitional forest, Tulear Province, -24.93683°, 46.62667°, alt 180 m, M.E. Irwin, F.D. Parker, R. Harin’Hala, 01.06.2003, (1w, CASC); **CASENT0393137**, collection code: BLF36793, Anosy Region, Parc National Andohahela, Col de Tanatana, -24.74969°, 46.84949°, alt 400 m, B.L.Fisher, F.A.Esteves et al., 03.09.2015, (1w, CASC); **CASENT0418656**, collection code: BLF05760, Mahafaly Plateau, 6.2 km 74° ENE Itampolo, -24.65361°, 43.99667°, alt 80 m, Fisher-Griswold Arthropod Team, 02.21.2002, (2w, CASC); **CASENT0418658**, collection code: BLF05760, Mahafaly Plateau, 6.2 km 74° ENE Itampolo, -24.65361°, 43.99667°, alt 80 m, Fisher-Griswold Arthropod Team, 02.21.2002, (2w, CASC); **CASENT0427196**, collection code: BLF06256, Parc National de Tsimanampetsotsa, Forêt de Bemanateza, 20.7 km 81° E Efoetse, 23.0 km 131° SE Beheloka, -23.99222°, 43.88067°, alt 90 m, Fisher-Griswold Arthropod Team, 03.22.2002, (1w, CASC); **CASENT0427197**, collection code: BLF06256, Parc National de Tsimanampetsotsa, Forêt de Bemanateza, 20.7 km 81° E Efoetse, 23.0 km 131° SE Beheloka, -23.99222°, 43.88067°, alt 90 m, Fisher-Griswold Arthropod Team, 03.22.2002, (1w, CASC); **CASENT0440935**, collection code: BLF06159, Parc National de Tsimanampetsotsa, Mitoho Cave, 6.4 km 77° ENE Efoetse, 17.4 km 170° S Beheloka, -24.04722°, 43.75317°, alt 40 m, Fisher-Griswold Arthropod Team, 03.18.2002, (2w, CASC); **CASENT0445101**, collection code: BLF06320, Parc National de Tsimanampetsotsa, Forêt de Bemanateza, 20.7 km 81° E Efoetse, 23.0 km 131° SE Beheloka, -23.99222°, 43.88067°, alt 90 m, Fisher-Griswold Arthropod Team, 03.22.2002, (1w, CASC); **CASENT0445102**, collection code: BLF06320, Parc National de Tsimanampetsotsa, Forêt de Bemanateza, 20.7 km 81° E Efoetse, 23.0 km 131° SE Beheloka, -23.99222°, 43.88067°, alt 90 m, Fisher-Griswold Arthropod Team, 03.22.2002, (1w, CASC); **CASENT0445936**, collection code: BLF05502, Réserve Spéciale de Cap Sainte Marie, 12.3 km 262° W Marovato, -25.58167°, 45.16833°, alt 200 m, Fisher-Griswold Arthropod Team, 02.11.2002, (2w, CASC); **CASENT0449258**, collection code: BLF05824, Mahafaly Plateau, 6.2 km 74° ENE Itampolo, -24.65361°, 43.99667°, alt 80 m, Fisher-Griswold Arthropod Team, 02.21.2002, (1w, CASC); **CASENT0449268**, collection code: BLF05833, Mahafaly Plateau, 6.2 km 74° ENE Itampolo, -24.65361°, 43.99667°, alt 80 m, Fisher-Griswold Arthropod Team, 02.21.2002, (1w, CASC); **CASENT0455014**, collection code: BLF06240, Parc National de Tsimanampetsotsa, Mitoho Cave, 6.4 km 77° ENE Efoetse, 17.4 km 170° S Beheloka, -24.04722°, 43.75317°, alt 40 m, Fisher-Griswold Arthropod Team, 37333, (2w, CASC); **CASENT0455831**, collection code: BLF06192, Parc National de Tsimanampetsotsa, Mitoho Cave, 6.4 km 77° ENE Efoetse, 17.4 km 170° S Beheloka, -24.04722°, 43.75317°, alt 40 m, Fisher-Griswold Arthropod Team, 03.18.2002, (2w, CASC);

##### Etymology.

The name (cingulatus) refers to the conspicuous dark brown transversal patch on the first gastral tergite of workers of this species.

##### Diagnosis in key.

**Description of workers.** Body color: yellow; brown. Body color pattern: concolorous, first gaster tergite darker. Absolute cephalic size: 459 [419, 515]. Cephalic length vs. maximum width of head capsule (CL/CWb): 1.37 [1.30, 1.45]. Postocular distance vs. cephalic length (PoOc/CL): 0.51 [0.49, 0.52]. Postocular sides of cranium contour frontal view orientation: converging posteriorly. Postocular sides of cranium contour frontal view shape: broadly convex. Vertex contour line in frontal view shape: straight. Vertex sculpture: main sculpture absent, ground sculpture areolate. Setal pits on head dorsum: surrounded by conspicuous foveae with well-demarcated margins. Diameter of foveae on head dorsum: 15-20 µm. Gena contour line in frontal view shape: straight. Genae contour from anterior view orientation: converging. Gena sculpture: rugoso-reticulate with areolate ground sculpture; ground sculpture areolate, main sculpture absent. Concentric carinae laterally surrounding antennal foramen: present. Eye length vs. absolute cephalic size (EL/CS): 0.24 [0.22, 0.26]. Frontal carina distance vs. absolute cephalic size (FRS/CS): 0.31 [0.30, 0.33]. Longitudinal carinae on median region of frons: absent. Smooth median region on frons: absent. Antennomere count: 12. Scape length vs. absolute cephalic size (SL/CS): 0.63 [0.60, 0.65]. Median clypeal notch: present. Ground sculpture of submedian area of clypeus: present. Median carina of clypeus: absent. Metanotal depression: present. Dorsal region of mesosoma sculpture: ground sculpture areolate, main sculpture absent. Lateral region of pronotum sculpture: ground sculpture areolate, main sculpture absent. Mesopleuron sculpture: ground sculpture areolate, main sculpture absent. Metapleuron sculpture: ground sculpture areolate, main sculpture absent. Petiole width vs. absolute cephalic size (PEW/CS): 0.22 [0.21, 0.24]. Dorsal region of petiole sculpture: ground sculpture areolate, main sculpture absent. Postpetiole width vs. absolute cephalic size (PPW/CS): 0.32 [0.28, 0.35]. Dorsal region of postpetiole sculpture: ground sculpture areolate, main sculpture absent. Surface of first gastral tergite: continuously imbricate.

##### Distribution.

This species is known to occur in the spiny forests of the southern part of Madagascar from 40–400 m (Fig. 21). The known samples are collected from live stems.

#### Nesomyrmex edentatus

Taxon classificationAnimaliaHymenopteraFormicidae

Csősz & Fisher sp. n.

http://zoobank.org/FF26C1C3-2ADA-45F6-8825-AA60267D0304

**Figures 22–24.**
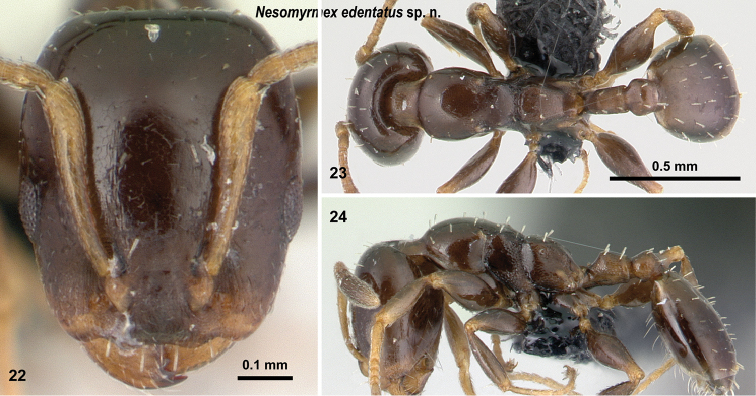
*Nesomyrmex edentatus* sp. n. holotype worker (CASENT0067086). Head in full-face view (**22**), dorsal view of the body (**23**), lateral view of the body (**24**).

**Figure 25.**
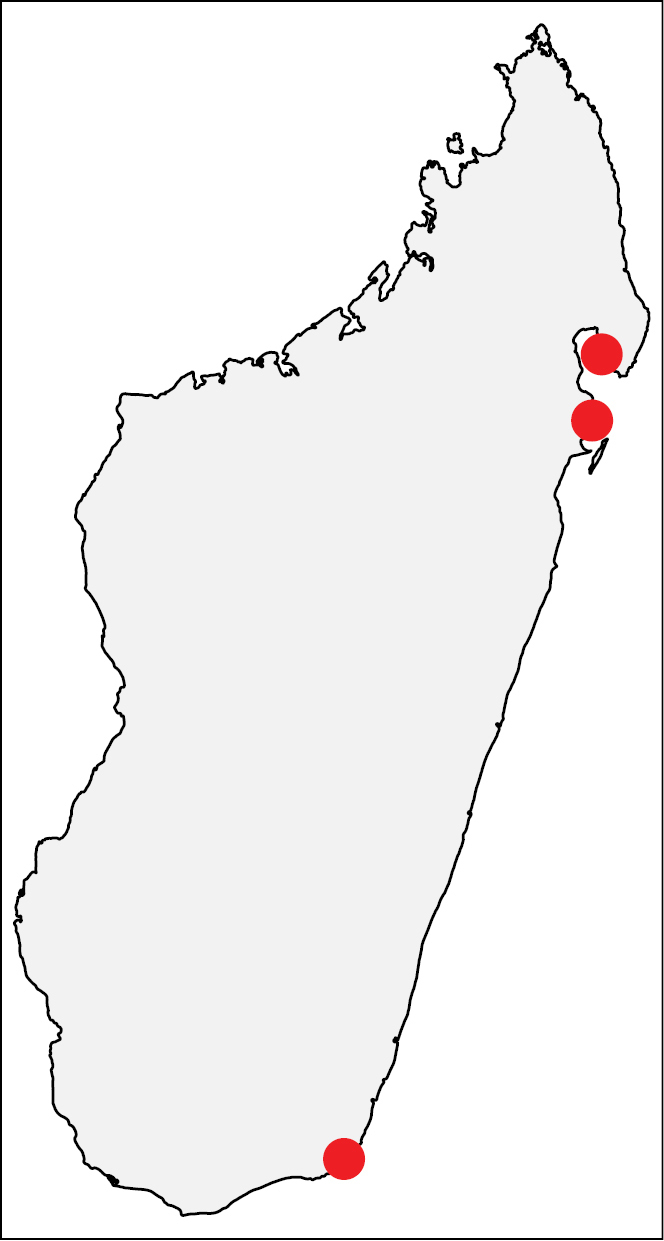
Geographic distribution of *Nesomyrmex edentatus* sp. n.. The known localities of *Nesomyrmex edentatus* sp. n. in Madagascar.

Figs 22–24 , 25 , Table 1

##### Type material investigated.

**Holotype: CASENT0067086**, collection code: BLF12553, Forêt d’Analava Mandrisy, 5.9 km 195ºAntanambe, -16.48567°, 49.847°, alt 10 m, B.L.Fisher et al., 11.13.2005, (1w, CASC, CASENT0067086);

**Paratypes: CASENT0004811**, collection code: ANTC1302, P.N. Masoala, 40 km 154° SSE Maroantsetra, -15.72667°, 49.95667°, alt 150 m, A. Dejean et al., 10.14.2001, (1w, CASC); **CASENT0121563**, collection code: BLF15678, Forêt Mandena 8.5 km N Tolagnaro, -24.95267°, 47.0025°, alt 20 m, B.L.Fisher et al., 12.05.2006, (1w, CASC);

##### Etymology.

The name (edentatus) refers to the absence of propodeal teeth in this species.

##### Diagnosis in key.

**Description of workers.** Body color: black. Body color pattern: concolorous. Absolute cephalic size: 546 [535, 560]. Cephalic length vs. maximum width of head capsule (CL/CWb): 1.20 [1.19, 1.21]. Postocular distance vs. cephalic length (PoOc/CL): 0.48 [0.47, 0.49]. Postocular sides of cranium contour frontal view orientation: converging posteriorly. Postocular sides of cranium contour frontal view shape: broadly convex. Vertex contour line in frontal view shape: straight. Vertex sculpture: main sculpture absent, ground sculpture imbricate, shiny. Setal pits on head dorsum: inconspicuous with obscure demarcation. Gena contour line in frontal view shape: straight. Genae contour from anterior view orientation: converging. Gena sculpture: rugoso-reticulate with areolate ground sculpture. Concentric carinae laterally surrounding antennal foramen: present. Eye length vs. absolute cephalic size (EL/CS): 0.25 [0.23, 0.26]. Frontal carina distance vs. absolute cephalic size (FRS/CS): 0.30 [0.29, 0.31]. Longitudinal carinae on median region of frons: absent. Smooth median region on frons count: present. Antennomere count: 12. Scape length vs. absolute cephalic size (SL/CS): 0.66 [0.65, 0.67]. Median clypeal notch: present. Ground sculpture of submedian area of clypeus: present. Median carina of clypeus: absent. Metanotal depression: present. Dorsal region of mesosoma sculpture: imbricate, main sculpture absent. Lateral region of pronotum sculpture: imbricate, main sculpture absent. Mesopleuron sculpture: ground sculpture areolate, main sculpture absent. Metapleuron sculpture: ground sculpture areolate, main sculpture absent. Petiole width vs. absolute cephalic size (PEW/CS): 0.20 [0.20, 0.22]. Dorsal region of petiole sculpture: ground sculpture areolate, main sculpture absent. Postpetiole width vs. absolute cephalic size (PPW/CS): 0.28 [0.27, 0.28]. Dorsal region of postpetiole sculpture: ground sculpture areolate, main sculpture absent. Surface of first gastral tergite: imbricate sculpture absent.

##### Distribution.

This species is known to occur in the littoral rainforests of eastern coastal Madagascar (Fig. 25). All samples were collected via beating low vegetation.

#### Nesomyrmex flavigaster

Taxon classificationAnimaliaHymenopteraFormicidae

Csősz & Fisher sp. n.

http://zoobank.org/CBB5252E-4A1D-437C-ABC9-884F6BC99F48

**Figures 26–28.**
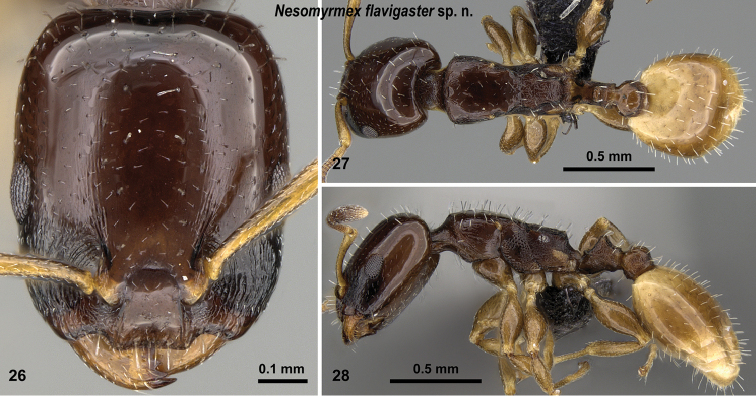
*Nesomyrmex flavigaster* sp. n. holotype worker (CASENT0453374). Head in full-face view (**26**), dorsal view of the body (**27**), lateral view of the body (**28**).

**Figure 29.**
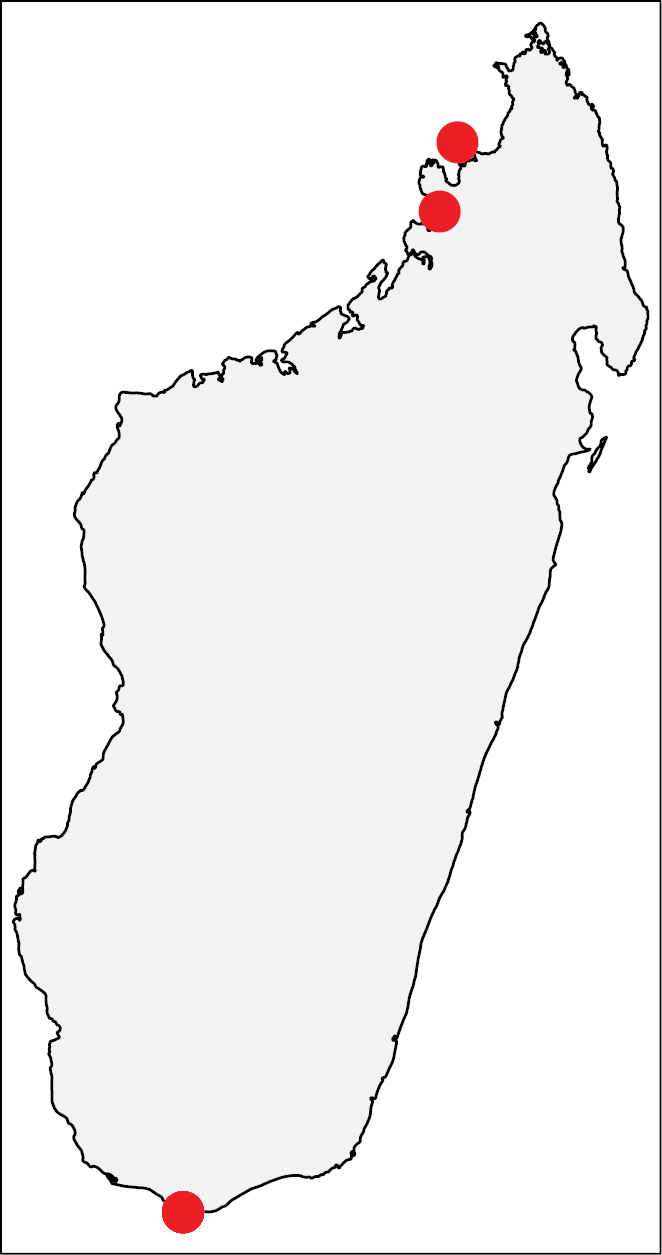
Geographic distribution of *Nesomyrmex flavigaster* sp. n.. The known localities of *Nesomyrmex flavigaster* sp. n. in Madagascar.

Figs 26–28 , 29 , Table 1

##### Type material investigated.

**Holotype: CASENT0453374**, collection code: BLF05530, Réserve Spéciale de Cap Sainte Marie, 12.3 km 262° W Marovato, -25.58167°, 45.16833°, alt 200 m, Fisher-Griswold Arthropod Team, 02.11.2002, (1w, CASC, CASENT0453374);

**Paratypes**: 25 workers from the same locality under CASENT codes: **CASENT0453367**, collection code: BLF05530, (1w, CASC); **CASENT0453368**, collection code: BLF05530, (1w, CASC); **CASENT0453369**, collection code: BLF05530, (1w, CASC); **CASENT0453370**, collection code: BLF05530, (1w, CASC); **CASENT0453371**, collection code: BLF05530, (1w, CASC); **CASENT0453372**, collection code: BLF05530, (1w, CASC); **CASENT0453373**, collection code: BLF05530, (1w, CASC); **CASENT0453376**, collection code: BLF05530, (1w, CASC); **CASENT0453377**, collection code: BLF05530, (1w, CASC); **CASENT0453378**, collection code: BLF05530, (1w, CASC); **CASENT0453379**, collection code: BLF05530, (1w, CASC); **CASENT0453380**, collection code: BLF05530, (1w, CASC); **CASENT0453381**, collection code: BLF05530, (1w, CASC); **CASENT0453382**, collection code: BLF05530, (1w, CASC); **CASENT0453383**, collection code: BLF05530, (1w, CASC); **CASENT0453384**, collection code: BLF05530, (1w, CASC); **CASENT0453385**, collection code: BLF05530, (1w, CASC); **CASENT0453386**, collection code: BLF05530, (1w, CASC); **CASENT0453387**, collection code: BLF05530, (1w, CASC); **CASENT0453388**, collection code: BLF05530, (1w, CASC); **CASENT0453389**, collection code: BLF05530, (2w, CASC); **CASENT0453390**, collection code: BLF05530, (3w, CASC, CASENT0453390).

##### Material examined.

**MADAGASCAR: CASENT0136889**, collection code: BLF20382, Nosy Be airport, -13.3138°, 48.31509°, alt 25 m, B.L.Fisher et al., 04.015.2008, (1w, CASC); **CASENT0453655**, collection code: BLF05644, Réserve Spéciale de Cap Sainte Marie, 12.3 km 262° W Marovato, -25.58167°, 45.16833°, alt 200 m, Fisher-Griswold Arthropod Team, 02.11.2002, (1w, CASC); **CASENT0453659**, collection code: BLF05644, Réserve Spéciale de Cap Sainte Marie, 12.3 km 262° W Marovato, -25.58167°, 45.16833°, alt 200 m, Fisher-Griswold Arthropod Team, 02.11.2002, (1w, CASC); **CASENT0453661**, collection code: BLF05526, Réserve Spéciale de Cap Sainte Marie, 12.3 km 262° W Marovato, -25.58167°, 45.16833°, alt 200 m, Fisher-Griswold Arthropod Team, 02.11.2002, (1w, CASC); **CASENT0453665**, collection code: BLF05526, Réserve Spéciale de Cap Sainte Marie, 12.3 km 262° W Marovato, -25.58167°, 45.16833°, alt 200 m, Fisher-Griswold Arthropod Team, 02.11.2002, (1w, CASC); **CASENT0453685**, collection code: BLF05586, Réserve Spéciale de Cap Sainte Marie, 12.3 km 262° W Marovato, -25.58167°, 45.16833°, alt 200 m, Fisher-Griswold Arthropod Team, 02.11.2002, (1w, CASC); **CASENT0453687**, collection code: BLF05586, Réserve Spéciale de Cap Sainte Marie, 12.3 km 262° W Marovato, -25.58167°, 45.16833°, alt 200 m, Fisher-Griswold Arthropod Team, 02.11.2002, (1w, CASC); **CASENT0477291**, collection code: BLF05504, Réserve Spéciale de Cap Sainte Marie, 12.3 km 262° W Marovato, -25.58167°, 45.16833°, alt 200 m, Fisher-Griswold Arthropod Team, 02.11.2002, (1w, CASC).

##### Etymology.

The name (flavigaster) refers to the light yellow gaster of this species, which is in sharp contrast to the dark brown color of the rest of the body.

##### Diagnosis in key.

**Description of workers.** Body color: dark brown; black. Body color pattern: body concolorous, gaster pale yellow. Absolute cephalic size: 629 [546, 740]. Cephalic length vs. maximum width of head capsule (CL/CWb): 1.23 [1.13, 1.29]. Postocular distance vs. cephalic length (PoOc/CL): 0.43 [0.41, 0.46]. Postocular sides of cranium contour frontal view orientation: converging posteriorly. Postocular sides of cranium contour frontal view shape: broadly convex. Vertex contour line in frontal view shape: straight. Vertex sculpture: main sculpture absent, ground sculpture absent, smooth and shiny. Setal pits on head dorsum: inconspicuous with obscure demarcation. Gena contour line in frontal view shape: convex. Genae contour from anterior view orientation: converging. Gena sculpture: ground sculpture areolate, main sculpture absent; ground sculpture areolate, main sculpture costulate. Concentric carinae laterally surrounding antennal foramen: present. Eye length vs. absolute cephalic size (EL/CS): 0.23 [0.21, 0.25]. Frontal carina distance vs. absolute cephalic size (FRS/CS): 0.34 [0.33, 0.36]. Longitudinal carinae on median region of frons: absent. Smooth median region on frons: present. Antennomere count: 12. Scape length vs. absolute cephalic size (SL/CS): 0.62 [0.59, 0.66]. Median clypeal notch: present. Ground sculpture of submedian area of clypeus: present. Median carina of clypeus: absent. Metanotal depression: present. Dorsal region of mesosoma sculpture: imbricate, main sculpture absent. Lateral region of pronotum sculpture: imbricate, main sculpture absent; inconspicious areolate ground sculpture, main sculpture dispersed costate. Mesopleuron sculpture: ground sculpture areolate, main sculpture absent. Metapleuron sculpture: ground sculpture areolate, main sculpture absent. Petiole width vs. absolute cephalic size (PEW/CS): 0.22 [0.20, 0.22]. Dorsal region of petiole sculpture: ground sculpture areolate, main sculpture dispersed rugose. Postpetiole width vs. absolute cephalic size (PPW/CS): 0.31 [0.29, 0.33]. Dorsal region of postpetiole sculpture: ground sculpture smooth, main sculpture absent. Surface of first gastral tergite: imbricate sculpture absent.

##### Distribution.

This species is known from Madagascar’s most remote spots (Fig. 29). The southern locality (Réserve Spéciale de Cap Sainte Marie, near Marovato), where it can be found in low vegetation of spiny forests in lowlands (200 m), seems the only known natural occurence of this species. The known northern samples were most probably introduced by humans in Nosy Be airport (urban garden) and Manerinerina (disturbed forest).

#### Nesomyrmex longiceps

Taxon classificationAnimaliaHymenopteraFormicidae

Csősz & Fisher sp. n.

http://zoobank.org/FA8BB966-98EF-403F-B7B1-3D62C36E3B1C

**Figures 30–32.**
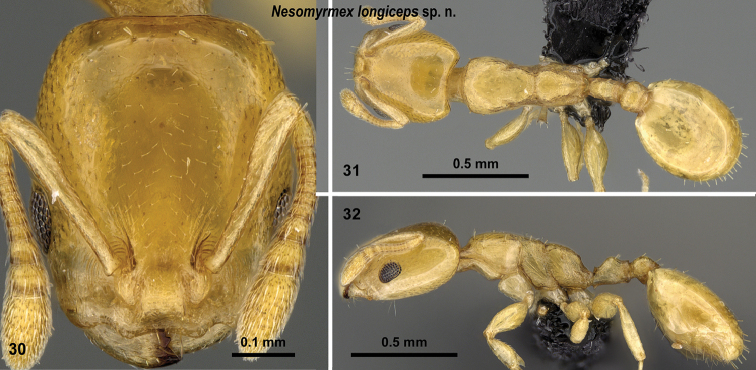
*Nesomyrmex longiceps* sp. n. holotype worker (CASENT0488528). Head in full-face view (**30**), dorsal view of the body (**31**), lateral view of the body (**32**).

**Figure 33.**
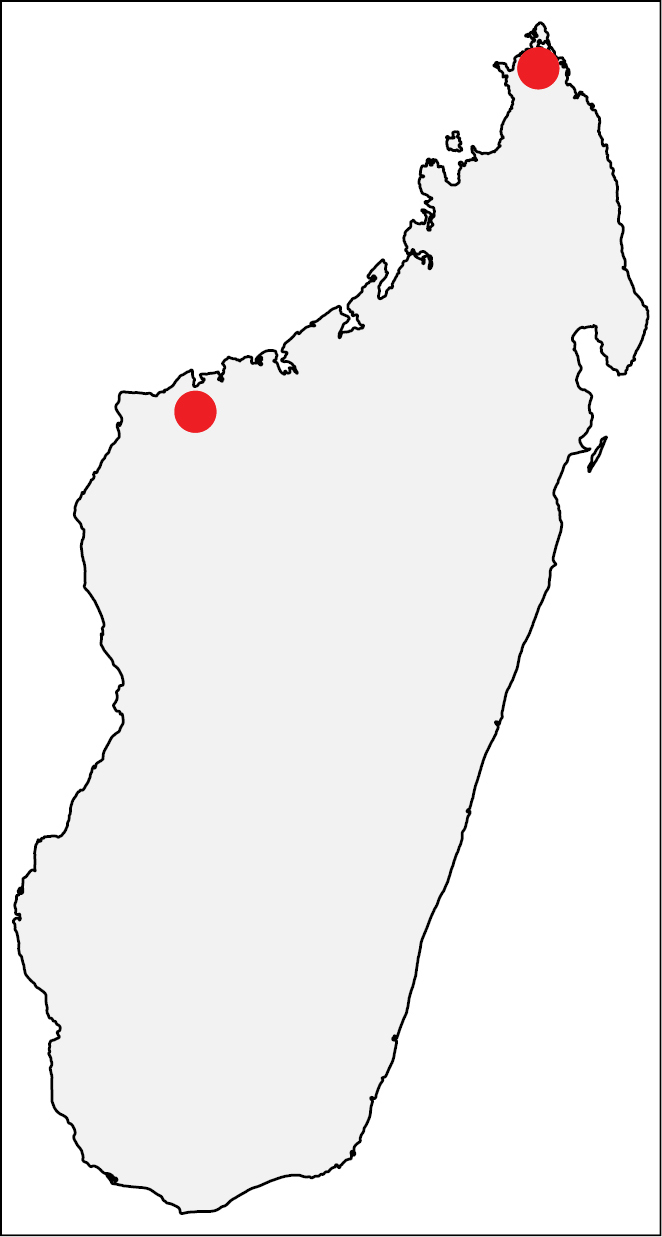
Geographic distribution of *Nesomyrmex longiceps* sp. n.. The known localities of *Nesomyrmex longiceps* sp. n. in Madagascar.

Figs 30–32 , 33 , Table 1

##### Type material investigated.

**Holotype: CASENT0488528**, collection code: BLF06584, Parc National de Namoroka, 16.9 km 317° NW Vilanandro, -16.40667°, 45.31°, alt 100 m, Fisher, Griswold et al., 11.12.2002, (1w, CASC, CASENT0488528);

**Paratype: CASENT0488529**, collection code: BLF06584, Parc National de Namoroka, 16.9 km 317° NW Vilanandro, -16.40667°, 45.31°, alt 100 m, Fisher, Griswold et al., 11.12.2002, (1w, CASC);

##### Material examined.

**MADAGASCAR: CASENT0400876**, collection code: BLF02660, Réserve Spéciale d’Ambre, 3.5 km 235° SW Sakaramy, -12.46889°, 49.24217°, alt 325 m, Fisher, Griswold et al., 01.26.2001, (1w, CASC); **CASENT0422685**, collection code: BLF02660, Réserve Spéciale d’Ambre, 3.5 km 235° SW Sakaramy, -12.46889°, 49.24217°, alt 325 m, Fisher, Griswold et al., 01.26.2001, (1w, CASC);

##### Etymology.

This name (longiceps) refers to the relatively elongated head of workers of this species.

##### Diagnosis in key.

**Description of workers.** Body color: yellow. Body color pattern: concolorous. Absolute cephalic size: 531 [478, 556]. Cephalic length vs. maximum width of head capsule (CL/CWb): 1.43 [1.42, 1.46]. Postocular distance vs. cephalic length (PoOc/CL): 0.44 [0.42, 0.45]. Postocular sides of cranium contour frontal view orientation: converging posteriorly. Postocular sides of cranium contour frontal view shape: broadly convex. Vertex contour line in frontal view shape: straight. Vertex sculpture: main sculpture absent, ground sculpture absent, smooth and shiny. Setal pits on head dorsum: inconspicuous with obscure demarcation. Gena contour line in frontal view shape: straight. Genae contour from anterior view orientation: converging. Gena sculpture: ground sculpture areolate, main sculpture costulate. Concentric carinae laterally surrounding antennal foramen: present. Eye length vs. absolute cephalic size (EL/CS): 0.22 [0.21, 0.23]. Frontal carina distance vs. absolute cephalic size (FRS/CS): 0.26 [0.25, 0.28]. Longitudinal carinae on median region of frons: absent. Smooth median region on frons: present. Antennomere count: 12. Scape length vs. absolute cephalic size (SL/CS): 0.63 [0.61, 0.65]. Median clypeal notch: present. Ground sculpture of submedian area of clypeus: present. Median carina of clypeus: absent. Metanotal depression: present. Dorsal region of mesosoma sculpture: imbricate, main sculpture absent. Lateral region of pronotum sculpture: ground sculpture areolate, main sculpture absent. Mesopleuron sculpture: ground sculpture areolate, main sculpture absent. Metapleuron sculpture: ground sculpture areolate, main sculpture absent. Petiole width vs. absolute cephalic size (PEW/CS): 0.19 [0.19, 0.19]. Dorsal region of petiole sculpture: ground sculpture areolate, main sculpture dispersed rugose. Postpetiole width vs. absolute cephalic size (PPW/CS): 0.27 [0.26, 0.28]. Dorsal region of postpetiole sculpture: ground sculpture smooth, main sculpture absent. Surface of first gastral tergite: imbricate sculpture absent.

##### Distribution.

This species is known to occur in northwestern Madagascar from 100–325 m (Fig. 33). The known samples were collected in tropical dry forests.

#### Nesomyrmex minutus

Taxon classificationAnimaliaHymenopteraFormicidae

Csősz & Fisher sp. n.

http://zoobank.org/786E5C5E-8DAA-4A60-BCEB-03E56B27CD3A

**Figures 34–36.**
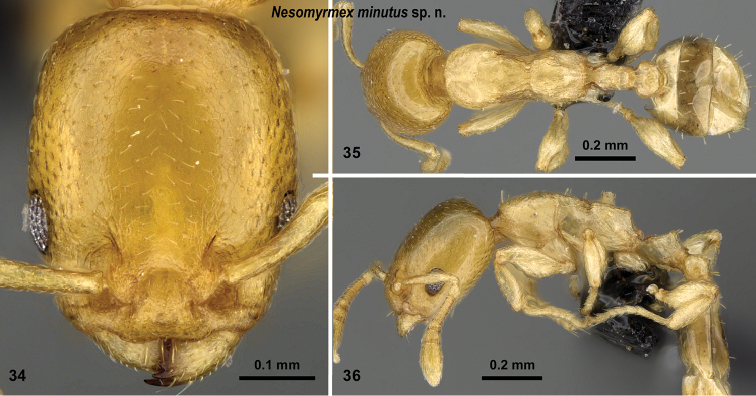
*Nesomyrmex minutus* sp. n. holotype worker (CASENT0071633). Head in full-face view (**34**), dorsal view of the body (**35**), lateral view of the body (**36**).

**Figures 37.**
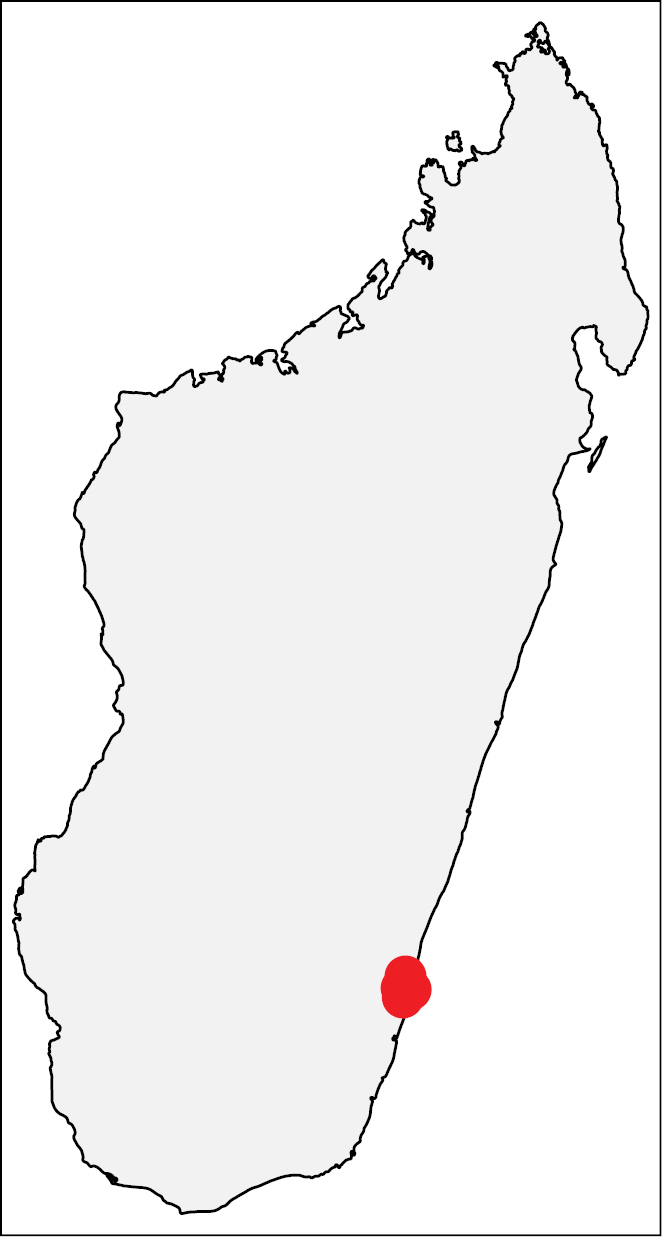
Geographic distribution of *Nesomyrmex minutus* sp. n.. The known localities of *Nesomyrmex minutus* sp. n. in Madagascar.

Figs 34–36 , 37 , Table 1

##### Type material investigated.

**Holotype: CASENT0071633**, collection code: BLF14082, Réserve Speciale Manombo 24.5 km 228° Farafangana, -23.01583°, 47.719°, alt 30 m, B.L. Fisher et al., 04.22.2006, (1w, CASC, CASENT0071633);

**Paratypes**: 21 workers, 16 gynes, and one male from the same locality under CASENT codes:

**CASENT0071611**, collection code: BLF14077, (1w, 1Q, CASC); **CASENT0071616**, collection code: BLF14066, (1w, CASC, CASENT0071616); **CASENT0071632**, collection code: BLF14076, (1w, 1Q, CASC); **CASENT0071639**, collection code: BLF14100, (1w, CASC); **CASENT0071640**, collection code: BLF14081, (1w, 1Q, CASC); **CASENT0071644**, collection code: BLF14073, (1w, 1Q, CASC); **CASENT0071646**, collection code: BLF14074, (1w, 1Q, CASC); **CASENT0071647**, collection code: BLF14071, (1w, 1Q, CASC); **CASENT0071650**, collection code: BLF14111, (1w, CASC); **CASENT0071657**, collection code: BLF14083, (1w, 1Q, CASC); **CASENT0071658**, collection code: BLF14067, (1w, CASC); **CASENT0071662**, collection code: BLF14064, (1w, 1Q, CASC); **CASENT0071667**, collection code: BLF14078, (1w, CASC); **CASENT0071668**, collection code: BLF14079, (1w, 1Q, CASC); **CASENT0071673**, collection code: BLF14072, (1w, 1Q, CASC); **CASENT0071676**, collection code: BLF14070, (1w, CASC); **CASENT0071714**, collection code: BLF14084, (1w, 1Q, CASC); **CASENT0071720**, collection code: BLF14069, (1w, CASC); **CASENT0071721**, collection code: BLF14080, (1w, 1Q, CASC); **CASENT0071725**, collection code: BLF14075, (1w, CASC); **CASENT0071656**, collection code: BLF14068, (1w, 1m, CASC); **CASENT0071675**, collection code: BLF14063, (1Q, CASC); **CASENT0071645**, collection code: BLF14073, (1Q, CASC); **CASENT0071636**, collection code: BLF14076, (1Q, CASC); **CASENT0071659**, collection code: BLF14083, (1Q, CASC);

##### Etymology.

The name (minutus) refers to the small tiny appearance of workers of this species.

##### Diagnosis in key.

**Description of workers.** Body color: yellow. Body color pattern: body concolorous, first gaster tergite darker. Absolute cephalic size: 469 [405, 530]. Cephalic length vs. maximum width of head capsule (CL/CWb): 1.30 [1.22, 1.36]. Postocular distance vs. cephalic length (PoOc/CL): 0.52 [0.51, 0.54]. Postocular sides of cranium contour frontal view orientation: converging posteriorly. Postocular sides of cranium contour frontal view shape: broadly convex. Vertex contour line in frontal view shape: straight. Vertex sculpture: main sculpture absent, ground sculpture imbricate, shiny. Setal pits on head dorsum: inconspicuous with obscure demarcation. Gena contour line in frontal view shape: convex. Genae contour from anterior view orientation: converging. Gena sculpture: rugoso-reticulate with areolate ground sculpture; ground sculpture areolate, main sculpture costulate. Concentric carinae laterally surrounding antennal foramen: present. Eye length vs. absolute cephalic size (EL/CS): 0.25 [0.22, 0.28]. Frontal carina distance vs. absolute cephalic size (FRS/CS): 0.36 [0.33, 0.39]. Longitudinal carinae on median region of frons: absent. Smooth median region on frons: present. Antennomere count: 12. Scape length vs. absolute cephalic size (SL/CS): 0.57 [0.53, 0.61]. Median clypeal notch: present. Ground sculpture of submedian area of clypeus: present. Median carina of clypeus: absent. Metanotal depression: present. Dorsal region of mesosoma sculpture: imbricate, main sculpture absent. Lateral region of pronotum sculpture: imbricate, main sculpture absent; inconspicious areolate ground sculpture, main sculpture dispersed costate. Mesopleuron sculpture: ground sculpture areolate, main sculpture absent. Metapleuron sculpture: ground sculpture areolate, main sculpture absent. Petiole width vs. absolute cephalic size (PEW/CS): 0.22 [0.20, 0.23]. Dorsal region of petiole sculpture: ground sculpture areolate, main sculpture absent. Postpetiole width vs. absolute cephalic size (PPW/CS): 0.33 [0.30, 0.35]. Dorsal region of postpetiole sculpture: ground sculpture smooth, main sculpture absent. Surface of the first gastral tergite: imbricate sculpture absent.

##### Distribution.

This species is known from rainforests of a single locality in the southeastern coast of Madagascar (Fig. 37), where it appears to be exclusively associated with the host plant *Macphersonia gracilis* (Sapindaceae). We know little about the ecology of the association. Unlike other tropical regions, very few ant plant associations are known from Madagascar. The only known ant assocations are from notheastern Madagascar, where three species in the plant genus *Gravesia* Naudin, 1851 (Melastomataceae) are often occupied by *Tetramorium silvicola* Hita Garcia & Fisher, 2014 and *Vitsika breviscapa* Bolton & Fisher, 2014.

#### Nesomyrmex punctaticeps

Taxon classificationAnimaliaHymenopteraFormicidae

Csősz & Fisher sp. n.

http://zoobank.org/0E95F739-536E-4ED2-9AEA-ED70E7620038

**Figures 38–40.**
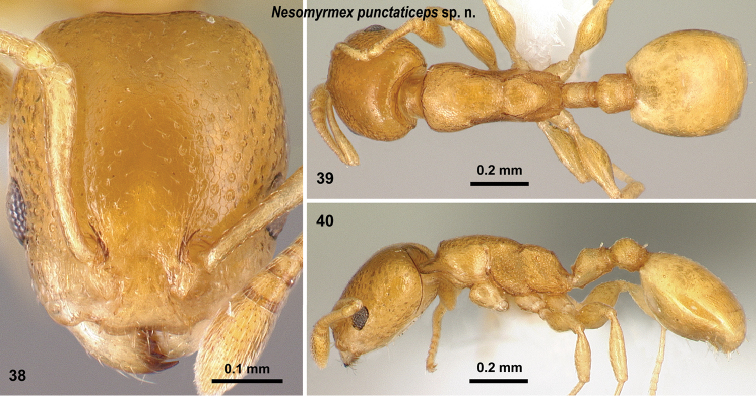
*Nesomyrmex punctaticeps* sp. n. holotype worker (CASENT0418448). Head in full-face view (**38**), dorsal view of the body (**39**), lateral view of the body (**40**).

**Figure 41.**
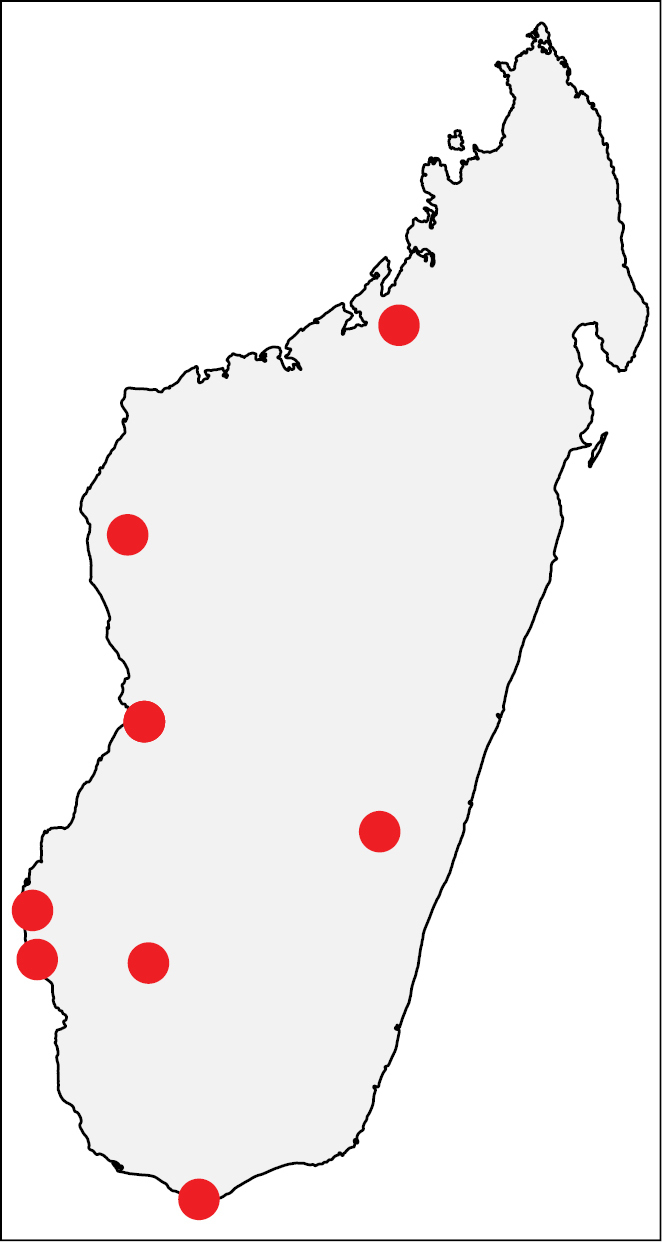
Geographic distribution of *Nesomyrmex punctaticeps* sp. n.. The known localities of *Nesomyrmex punctaticeps* sp. n. in Madagascar.

Figs 38–40 , 41 , Table 1

##### Type material investigated.

**Holotype: CASENT0418448**, collection code: BLF04604, Forêt de Kirindy, 15.5 km 64° ENE Marofandilia, -20.045°, 44.66222°, alt 100 m, Fisher-Griswold Arthropod Team, 11.28.2001, (1w, CASC, CASENT0418448);

**Paratypes**: four workers from the same locality under CASENT codes: **CASENT0418445**, collection code: BLF04604, (3w, CASC); **CASENT0418446**, collection code: BLF04604, (1w, CASC);

##### Material examined.

**MADAGASCAR: CASENT0002644**, collection code: BLF06141, Forêt de Beroboka, 5.9 km 131° SE Ankidranoka, -22.23306°, 43.36633°, alt 80 m, Fisher-Griswold Arthropod Team, 03.12.2002, (2w, CASC, CASENT0002644); **CASENT0004025**, collection code: BLF05637, 6.1 km 182° S Marovato, -25.58167°, 45.295°, alt 20 m, Fisher-Griswold Arthropod Team, 02.14.2002, (1w, CASC); **CASENT0156889**, collection code: BLF23048, Réserve forestière Beanka, 50.7 km E Maintirano, -17.88021°, 44.46877°, alt 140 m, B.L.Fisher et al., 10.30.2009, (2w, CASC); **CASENT0206506**, collection code: MG-33-03, Sofia Region,District of Port-Berger, Ambovomamy 20Km N of Port-Berger, Mahajanga, -15.45117°, 47.61333°, alt 86 m, M.F. Rin’ha, 01.21.2007, (1w, CASC); **CASENT0447338**, collection code: BLF05968, Forêt de Tsinjoriaky, 6.2 km 84° E Tsifota, -22.80222°, 43.42067°, alt 70 m, Fisher-Griswold Arthropod Team, 03.06.2002, (2w, CASC); **CASENT0484806**, collection code: BLF07511, Parc National de Zombitse, 19.8 km 84° E Sakaraha, -22.84333°, 44.71°, alt 770 m, Fisher, Griswold et al., 02.05.2003, (3w, CASC); **MCZENT0576250**, Ranomafana National Park, -21.32°, 47.39°, alt 900 m, W.E. Steiner, 01.02.1990, (1w, MCZC);

##### Etymology.

This name (punctaticeps) refers to conspicuously foveolate head dorsum of workers of this species.

##### Diagnosis in key.

**Description of workers.** Body color: yellow. Body color pattern: concolorous. Absolute cephalic size: 473 [424, 549]. Cephalic length vs. maximum width of head capsule (CL/CWb): 1.20 [1.14, 1.26]. Postocular distance vs. cephalic length (PoOc/CL): 0.49 [0.47, 0.51]. Postocular sides of cranium contour frontal view orientation: converging posteriorly. Postocular sides of cranium contour frontal view shape: broadly convex. Vertex contour line in frontal view shape: straight. Vertex sculpture: main sculpture absent, ground sculpture imbricate, shiny. Setal pits on head dorsum: surrounded by conspicuous foveae with well demarcated margins. Diameter of foveae on head dorsum: 10 µm. Gena contour line in frontal view shape: convex. Genae contour from anterior view orientation: converging. Gena sculpture: rugoso-reticulate with areolate ground sculpture. Concentric carinae laterally surrounding antennal foramen count: present. Eye length vs. absolute cephalic size (EL/CS): 0.24 [0.22, 0.26]. Frontal carina distance vs. absolute cephalic size (FRS/CS): 0.33 [0.32, 0.35]. Longitudinal carinae on median region of frons: absent. Smooth median region on frons: present. Antennomere count: 12. Scape length vs. absolute cephalic size (SL/CS): 0.66 [0.62, 0.70]. Median clypeal notch: present. Ground sculpture of submedian area of clypeus: present. Median carina of clypeus: absent. Metanotal depression: present. Dorsal region of mesosoma sculpture: ground sculpture areolate, main sculpture absent. Lateral region of pronotum sculpture: ground sculpture areolate, main sculpture absent. Mesopleuron sculpture: ground sculpture areolate, main sculpture absent. Metapleuron sculpture: ground sculpture areolate, main sculpture absent. Petiole width vs. absolute cephalic size (PEW/CS): 0.22 [0.21, 0.24]. Dorsal region of petiole sculpture: ground sculpture areolate, main sculpture absent. Postpetiole width vs. absolute cephalic size (PPW/CS): 0.34 [0.30, 0.36]. Dorsal region of postpetiole sculpture: ground sculpture areolate, main sculpture absent. Surface of the first gastral tergite: incontinuously imbricate.

##### Distribution.

This species is widely distributed in Madagascar’s tropical dry forests and spiny forests (Fig. 41) in lower elevations between 20–770 m. Only one sample is known to be collected from rainforest of Ranomafana National Park in higher elevation (900 m). The known samples were collected by beating low vegetation.

#### Nesomyrmex sellaris

Taxon classificationAnimaliaHymenopteraFormicidae

Csősz & Fisher sp. n.

http://zoobank.org/C87A166F-4CC1-4A74-AA7A-39CDE98104AC

**Figures 42–44.**
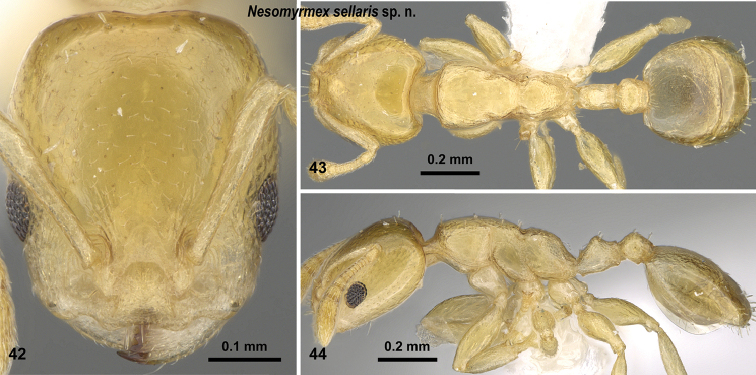
*Nesomyrmex sellaris* sp. n. holotype worker (CASENT0428261). Head in full-face view (**42**), dorsal view of the body (**43**), lateral view of the body (**44**).

**Figure 45.**
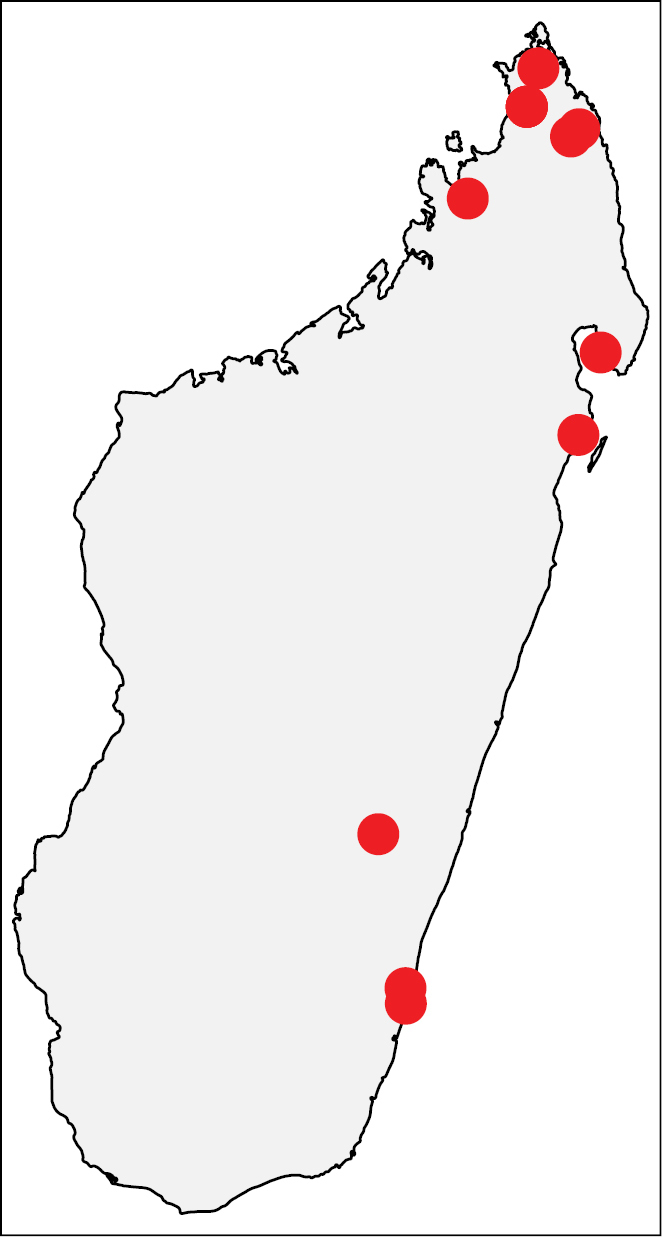
Geographic distribution of *Nesomyrmex sellaris* sp. n.. The known localities of *Nesomyrmex sellaris* sp. n. in Madagascar.

Figs 42–44 , 45 , Table 1

##### Type material investigated.

**Holotype: CASENT0428261**, collection code: BLF02859, Réserve Spéciale de l’Ankarana, 22.9 km 224° SW Anivorano Nord, -12.90889°, 49.10983°, alt 80 m, Fisher, Griswold et al., 02.10.2001, (1w, CASC, CASENT0428261);

**Paratypes**: four workers from the same locality under CASENT codes: **CASENT0422623**, collection code: BLF02859, (1w, CASC); **CASENT0422624**, collection code: BLF02859, (1w, CASC); **CASENT0428262**, collection code: BLF02859, (1w, CASC); **CASENT0422621**, collection code: BLF2859, (1w, CASC);

##### Material examined.

**MADAGASCAR: CASENT0004813**, collection code: ANTC1302, P.N. Masoala, 40 km 154° SSE Maroantsetra, -15.72667°, 49.95667°, alt 150 m, A. Dejean et al., 10.14.2001, (2w, CASC); **CASENT0053591**, collection code: MA-02-09B-53, radio tower, Ranomafana National Park, Fianarantsoa Prov., -21.25083°, 47.40717°, alt 1130 m, M. Irwin, R. Harin’Hala, 02.18.2003, (1w, CASC); **CASENT0068333**, collection code: BLF12740, Res. Ambodiriana, 4.8 km 306°Manompana, along Manompana river, -16.67233°, 49.70117°, alt 125 m, B.L.Fisher et al., 11.18.2005, (1w, CASC); **CASENT0070953**, collection code: BLF13926, Réserve Forestière d’Agnalazaha, Mahabo, 42.9 km 215° Farafangana, -23.19383°, 47.723°, alt 20 m, B.L. Fisher et al., 04.19.2006, (1w, CASC); **CASENT0071722**, collection code: BLF14065, Réserve Speciale Manombo 24.5 km 228° Farafangana, -23.01583°, 47.719°, alt 30 m, B.L. Fisher et al., 04.22.2006, (1w, CASC); **CASENT0189193**, collection code: BLF01998, R.S. Manongarivo, 10.8 km 229° SW Antanambao, -13.96167°, 48.43333°, alt 400 m, B.L.Fisher, 11.08.1998, (1w, CASC); **CASENT0262171**, collection code: MG-58-30, SAVA Region,Distric of Vohemar, Antsahabelela Rain Forest,9 km SW of Daraina, -13.2505°, 49.61667°, alt 182 m, Mike, Rin’ha, 04.27.2011, (1w, CASC); **CASENT0422618**, collection code: BLF02660, Réserve Spéciale de l’Ankarana, 22.9 km 224° SW Anivorano Nord, -12.90889°, 49.10983°, alt 80 m, Fisher, Griswold et al., 02.10.2001, (1w, CASC); **CASENT0422620**, collection code: BLF02660, Réserve Spéciale d’Ambre, 3.5 km 235° SW Sakaramy, -12.46889°, 49.24217°, alt 325 m, Fisher, Griswold et al., 01.26.2001, (1w, CASC); **CASENT0494407**, collection code: BLF09906, Forêt de Bekaraoka, 6.8 km 60° ENE Daraina, -13.16667°, 49.71°, alt 150 m, B.L.Fisher, 12.07.2003, (1w, CASC, CASENT0494407);

##### Etymology.

The name (sellaris) refers to the deep mesopropodeal depression in workers resembling a saddle (=sella Lat.).

##### Diagnosis in key.

**Description of workers.** Body color: yellow. Body color pattern: concolorous. Absolute cephalic size: 449 [410, 557]. Cephalic length vs. maximum width of head capsule (CL/CWb): 1.18 [1.13, 1.25]. Postocular distance vs. cephalic length (PoOc/CL): 0.51 [0.49, 0.53]. Postocular sides of cranium contour frontal view orientation: converging posteriorly. Postocular sides of cranium contour frontal view shape: broadly convex. Vertex contour line in frontal view shape: straight. Vertex sculpture: main sculpture absent, ground sculpture imbricate, shiny. Setal pits on head dorsum: surrounded by conspicuous foveae with well demarcated margins. Diameter of foveae on head dorsum: 10–15 µm. Gena contour line in frontal view shape: straight. Genae contour from anterior view orientation: converging. Gena sculpture: rugoso-reticulate with areolate ground sculpture. Concentric carinae laterally surrounding antennal foramen: present. Eye length vs. absolute cephalic size (EL/CS): 0.24 [0.23, 0.26]. Frontal carina distance vs. absolute cephalic size (FRS/CS): 0.29 [0.27, 0.30]. Longitudinal carinae on median region of frons: absent. Smooth median region on frons: present. Antennomere count: 12. Scape length vs. absolute cephalic size (SL/CS): 0.69 [0.65, 0.72]. Median clypeal notch: present. Ground sculpture of submedian area of clypeus: present. Median carina of clypeus: absent. Metanotal depression: present. Dorsal region of mesosoma sculpture: imbricate, main sculpture absent. Lateral region of pronotum sculpture: imbricate, main sculpture absent; inconspicious areolate ground sculpture, main sculpture dispersed costate. Mesopleuron sculpture: ground sculpture areolate, main sculpture absent. Metapleuron sculpture: ground sculpture areolate, main sculpture absent. Petiole width vs. absolute cephalic size (PEW/CS): 0.21 [0.20, 0.23]. Dorsal region of petiole sculpture: ground sculpture areolate, main sculpture absent. Postpetiole width vs. absolute cephalic size (PPW/CS): 0.29 [0.26, 0.31]. Dorsal region of postpetiole sculpture: ground sculpture smooth, main sculpture absent. Surface of the first gastral tergite: incontinuously imbricate; continuously imbricate.

##### Distribution.

This species is widely distributed in East Madagascar (Fig. 45). The known samples were collected in diverse habitats from tropical dry forests to rainforests, typically at lower elevation from 20–1130 m.

## Supplementary Material

XML Treatment for Nesomyrmex brevicornis

XML Treatment for Nesomyrmex brunneus

XML Treatment for Nesomyrmex cingulatus

XML Treatment for Nesomyrmex edentatus

XML Treatment for Nesomyrmex flavigaster

XML Treatment for Nesomyrmex longiceps

XML Treatment for Nesomyrmex minutus

XML Treatment for Nesomyrmex punctaticeps

XML Treatment for Nesomyrmex sellaris
